# Identification of Key Genes Associated With Early Calf-Hood Nutrition in Subcutaneous and Visceral Adipose Tissues by Co-Expression Analysis

**DOI:** 10.3389/fvets.2022.831129

**Published:** 2022-05-10

**Authors:** Cuili Pan, Chaoyun Yang, Yanfen Ma, Hui Sheng, Zhaoxiong Lei, Shuzhe Wang, Honghong Hu, Xue Feng, Junxing Zhang, Yun Ma

**Affiliations:** Key Laboratory of Ruminant Molecular and Cellular Breeding, School of Agriculture, Ningxia University, Yinchuan, China

**Keywords:** adipose tissue, diet, WGCNA, hub genes, nutrition

## Abstract

**Background:**

Substantive evidence has confirmed that nutrition state is associated with health risk and the onset of pubertal and metabolic profile. Due to heterogeneity, adipose tissues in different anatomical positions tend to show various metabolic mechanisms for nutrition. To date, the complicated molecular mechanisms of early calf-hood nutrition on bovine adipose tissue are still largely unknown. This study aimed to identify key genes and functionally enriched pathways associated with early calf-hood nutrition in visceral and subcutaneous adipose tissue.

**Results:**

The RNA-seq data of visceral and subcutaneous adipose tissues of calves feeding on low and high dietary nutrition for more than 100 days were downloaded and analyzed by weighted gene co-expression network analysis (WGCNA). Two modules that positively associated with a low plane of nutrition diet and two modules with a high plane of nutrition diet were identified in the subcutaneous adipose tissue. The blue and yellow modules, most closely associated with low and high nutrition, were selected for the functional enrichment analysis and exploration of hub genes. The results showed that genes in the blue module were significantly enriched in pathways that related to fat metabolism, reproduction, and cell communication. Genes in the yellow module were enriched in pathways related to fat metabolism, reproduction, cell proliferation, and senescence. Meanwhile, the blue and brown modules in visceral adipose tissue were most closely associated with low and high nutrition, respectively. Notably, genes of the blue module were significantly enriched in pathways related to substance metabolism, and genes in the brown module were significantly enriched in energy metabolism and disease pathways. Finally, key genes in subcutaneous adipose tissue for low nutrition (*PLCG1, GNA11*, and *ANXA5*) and high nutrition (*BUB1B, ASPM, RRM2, PBK, NCAPG*, and *MKI67*), and visceral adipose tissue for low nutrition (*RPS5, RPL4, RPL14*, and *RPLP0*) and high nutrition (*SDHA* and *AKT1*) were obtained and verified.

**Conclusion:**

The study applied WGCNA to identify hub genes and functionally enriched pathways in subcutaneous and visceral adipose tissue and provided a basis for studying the effect of early calf-hood nutrition on the two adipose tissue types.

## Background

Improving economic benefit is the intrinsic power of sustainable development in the breeding industry. Nutritional status has been shown to affect many economically important traits, such as lifetime growth, feed intake, carcass composition, health condition, and reproductive development (1–3). For instance, the supplement of a molasses-based liquid feed in high-straw diets improved the intake and consistency of nutrients consumed during the dry period and early lactation, as well as possibly promoting rumen health during the transition period (4). In Holstein steers, a high-density diet improves growth performance and beef yield, whereas it has a negative effect on their serum metabolism and visceral morphology (5). A serious energy deficit suppresses the secretion of gonadotrophins, IGF-I, plasmic insulin, and progesterone, thus affecting fertility indicators (6, 7). Meanwhile, diet affects histotroph proteomes of embryo pre-implantation and understanding the impact may help to improve the conception rates (8). In bull calves of dairy breeds, a high level of early calf-hood nutrition is beneficial for pubertal onset, which is advantageous to facilitating early semen collection (9, 10).

Adipose tissue, as a vital node in the inter-organ crosstalk, could mediate the regulation of metabolism in multiple organs and tissues. Meanwhile, the nutritional state affects lipid metabolism and adipokines production, thus regulating a wide range of biological processes (9, 11). For instance, lecithin supplementation improved the meat quality in broilers by affecting the lipid metabolism and microbiota (12). Subcutaneous adipose tissue plays a significant role in energy storage and releases to balance expenditure and intake. The concentration of both leptin and adiponectin was higher in subcutaneous adipose tissue compared with visceral adipose (13). Low-fiber/high-starch diets and/or supplying lipids rich in polyunsaturated fatty acids induce milk fat depression (MFD) and increased the transcription of adipogenic genes in the subcutaneous adipose tissue in cows (14). In non-lactating and non-pregnant Holstein cows, excess energy intake increased adipose tissue mass by promoting the transcription of key genes associated with adipogenesis, lipogenesis, and insulin signaling (15). Omental, mesenteric, and perirenal adipose tissue masses were larger in non-lactating dairy cows fed high energy than low energy, and body condition score (BCS) was correlated with the masses of omental (*r* = 0.57), mesenteric (*r* = 0.59), and perirenal (*r* = 0.72) adipose tissue (16). Diets with a high or low proportion of concentrate and the supplementation of niacin regulated metabolic conversion from late gestation to early lactation by affecting the secretion of adipokines in subcutaneous and visceral adipose tissues of dairy cows (17). Due to the heterogeneity, it is speculated that subcutaneous and visceral adipose tissues might exhibit a different metabolic response to nutritional status. However, to our best knowledge, the exact and complicated function and regulatory mechanism are still unclear.

Weighted gene co-expression network analysis can be used to identify modules, associate them with external information (trait, pathway, SNP, or QTL) and measure the relationship between modules or genes and the studied traits (18). To date, WGCNA has been widely used to explore co-expression modules and genes that are associated with diseases, such as type I diabetes (19), Sjögren's Syndrome (20), rheumatoid arthritis (21), and bronchopulmonary dysplasia (22). There is also a possibility to utilize it to evaluate complex traits and correlate genes and phenotype in cattle. Our study aims to use WGCNA to explore the gene co-expression network of early calf-hood nutrition on visceral and subcutaneous adipose tissue. In addition, for the genes in the most related module to the high and low plane of nutrition, Kyoto Encyclopedia of Genes and Genomes (KEGG) pathway and gene ontology (GO) analysis were conducted to explore their potential functions. An expression and receiver operating characteristic (ROC) curve analysis for hub genes were conducted to preliminarily verify the accuracy of the selection.

## Methods

### Data Collection and Pre-processing

The RNA-seq raw data of the two datasets were downloaded from the Sequence Read Archive (SRA) database (https://www.ncbi.nlm.nih.gov/sra/). The detailed experimental and phenotypical information of the two datasets was included in Supplementary Table 1. Nineteen Holstein-Friesian bull calves of similar status (with a mean age of 19 (±8.2) days and bodyweight of 47.5 (±5.3) kg) were retrieved from the dataset PRJNA382633. The calves were assigned to different diets (high or low plane of nutrition) under the same feeding management conditions. The dietary components could be referred to in the previous study (9). The feeding process under the condition of high and low dietary nutrient levels is as follows. Before weaning, the high nutrition group (*n* = 10) was offered a 1,200 g milk replacer in 8 L water (15% solids) daily, together with concentrate *ad libitum*. The low nutrition group (*n* = 9) was allocated 500 g milk replacer in 4 L water (12.5% solids) daily, together with a maximum of 1 kg of concentrate. After weaning, the high nutrition group was offered *ad libitum* concentrates, while the low nutrition group was offered 1 kg concentrate daily. At a mean age of 126 (±1.1) days, the subcutaneous adipose samples were obtained from the flank of the carcass for subsequent RNA sequencing.

A total of 29 Angus × Holstein-Friesian heifer calves with a mean age of 19 (±4) days and bodyweight of 51.2 (±7.8) kg were retrieved from the dataset PRJNA664093. The calves were assigned to two groups and fed on diets with a high or low plane of nutrition under the same feeding management conditions (10). The composition and chemical analysis of the offered reconstituted milk replacer and concentrate were provided in detail in the previous study (23). The reconstituted milk replacer was further configured as a liquid that contained 15% solid (20% fat and 26% protein), which was used to feed the calves according to the following standards. The high nutrition group (*n* = 14) was offered 10 L reconstituted milk replacer for 30 days; the 10 L reconstituted milk replacer was gradually reduced to 6 L during the following 5 days; then, 6 L reconstituted milk replacer was offered for 7 days; the 6 L reconstituted milk replacer was gradually reduced to 0 L during the final 14 days. The low nutrition group (*n* = 15) was offered a 4 L reconstituted milk replacer for 50 days, and was reduced to 0 L during the following 7 days. In addition, the high nutrition group was offered concentrate *ad libitum*, while the low nutrition group was offered restrained concentrate at a maximum of 1 kg per day during the week of weaning. At a mean age of 145 (±3) days, the visceral adipose samples were obtained from the same site within the omental fraction for subsequent RNA sequencing.

To obtain the consistent expression matrix, we used the same pipeline as follows to quantify the gene expression of the two datasets. The sequencing quality was checked using FastQC (version 0.11.9), and quality control of raw sequence data was performed using the Trim Galore (version 0.6.6). Clean reads were then mapped to the *Bos taurus* genome reference using Hisat2 (version 2.2.1), and the expression quantity of all genes was calculated using featureCounts (version 2.0.1).

### Construction of Co-expression Network

The WGCNA package was employed to construct gene co-expression networks in R (version 4.1.1) (18). The expression matrix was adjusted and normalized by log transformation using the normalizeBetweenArrays function of limma (R package). The top 8,000 genes were selected for subsequent analysis according to the median absolute deviation. The pickSoftThreshold function was used to choose an appropriate softthresholding power value (β) and obtain a scale-free topological network. In this study, we screened out the appropriate β value when the degree of independence reached 0.8. A weighted adjacency matrix was created, defined as A_mn_ = |cor (x_m_, x_n_)|^β^ (m and n represent two different genes, x_m_ and x_n_ are their respective expression values, and A_mn_ represents the Pearson's correlation coefficient). The one-step approach was adopted to build the network using the blockwiseModules function. The gene connectivity is defined as the sum of its adjacency with all other genes in the network. To measure the gene connectivity, the adjacent matrix was transformed into a topological overlap matrix (TOM). Hierarchical clustering was performed to divide modules with a minimum cluster size of 50 genes. Highly similar modules were merged with 0.25 as the cut height threshold.

### Identification of Modules Significantly Associated With Nutritional Levels

Module eigengene (ME) was defined as the first principal component of a module. It represents the single characteristic expression profile of the whole genes in the module and could give an idea of their expression patterns. To identify the modules and genes related to nutritional levels, we further associated the modules with phenotypic information. The correlation between MEs and nutritional levels in subcutaneous and visceral adipose tissues was evaluated by the Pearson's correlation test with *p* < 0.05 as the cut-off. The module most significantly related to nutritional levels in each fat type was considered the key module and subjected to further analysis.

### Identification of Hub Genes

Gene significance (GS) was defined as the correlation between gene expression and a specific phenotype and could be calculated by the equation GS_m_ = |cor (x_m_, T)|, where x_m_ is the expression of gene m, and T is a sample trait. Meanwhile, module membership (MM) was defined as the association between gene expression and each ME and could be quantified by the equation = |cor (x_m_, E^n^)|, where x_m_ is the expression of gene m, and E^n^ is the ME of module n. In this study, we identified the potential hub genes based on their GS value for the trait and MM value in the module. Subsequently, the functional protein association networks (PPI) analysis was performed on the STRING website (https://string-db.org/, version 11.5) (24). Moreover, four algorithms (MNC, EPC, Betweenness, and Degree) of the cytohubba plug-in were adopted to calculate the hub proteins using Cytoscape software (version = 3.8.2) (25). The overlap of the above results was defined as key genes, and the VennDiagram package in R was used to draw the Venn diagram.

### Functional Enrichment Analysis

To further explore genes in the interested modules, functional enrichment analysis was performed using the R package clusterProfiler (26). Specifically, the functions enrichGO and enrichKEGG were used for GO and KEGG analysis. The org.Bt.eg.db package (https://bioconductor.org/packages/release/data/annotation/html/org.Bt.eg.db.html) was used for the annotation and conversion of bovine genes in R software. Finally, the top 10 KEGG pathways and GO terms were identified for visualization.

### Gene Set Enrichment Analysis

Gene set enrichment analysis (GSEA) was conducted to further interpret the genome-wide expression profiles and explore the pathways related to nutrition levels in adipose tissues. The log2 fold change (FC) values of all genes were used as the input list. For single gene GSEA, the correlation between the interested gene and the other genes was calculated, and the obtained correlation matrix was used as the input list. ClusterProfiler and GSEABase package of the R software were utilized to conduct GSEA. The c2.cp.kegg.v7.1.symbols.gmt in Molecular Signatures Database (MSigDB) was selected as the reference gene set, and a *P*-value <0.05 was chosen as the cut-off criteria.

### Gene Set Variation Analysis

Gene set variation analysis (GSVA) is also a gene set enrichment method for assessing the variation in the pathway in an unsupervised manner. It transformed the expression matrix into a pathway matrix using GSEABase and GSVA packages, and the significant pathways were obtained by differential expression analysis using the limma package of R. The pheatmap package was employed to display the results.

### Differential Expression Validation and Efficacy Evaluation of Hub Genes

The hub genes were further validated by differential expression pattern analysis between the high and low planes of nutrition in the subcutaneous and visceral adipose tissue. The limma R package was utilized to screen the differentially expressed genes (DEGs) with *P*-value <0.05 and log2 (FC) ≥0.585 (|FC| ≥ 1.5). The ggplot2 package of R was used to draw the volcano plots. Meanwhile, the Wilcoxon test was also used to calculate the difference significance and visualize it in the R package ggpubr. In addition, the ROC curve was plotted and the area under the ROC curve (AUC) was calculated with the “pROC” package to evaluate the capability of selected genes to distinguish the high and low planes of nutrition (27).

### Statistical Analysis

The statistical significance was analyzed using a non-parametric test based on the characteristics of data distribution. All the analyses were performed in R software (version 4.2.3) and the statistical significance was selected as *P* < 0.05.

## Results

### Construction of Co-expression Networks

The two datasets (PRJNA382633 and PRJNA664093) containing high and low planes of nutrition were selected for WGCNA analysis to unearth modules and hub genes specific to nutrition levels in subcutaneous and visceral adipose tissues. Cluster analysis showed that samples of the same nutrition levels were classified into a group in subcutaneous adipose tissue (Supplementary Figure 1), indicating it was the main reason that caused the differences in gene expression. While in visceral adipose tissue, there were seven samples (the gray clade) that could not be separated (Supplementary Figure 1). The soft-thresholding power was determined when the correlation coefficient threshold reached 0.8 and co-expression networks were constructed subsequently (Supplementary Figures 2A, 3A).

A total of 20 co-expression modules from the two projects were identified *via* WGCNA analysis (Supplementary Table 2). In dataset PRJNA382633, the module containing most genes was the turquoise one (1,968 genes; account for 31.8%), followed by the blue module (1,397 genes; account for 22.5%), and the brown module (716 genes; account for 11.5%) (Supplementary Figure 2C). In dataset PRJNA664093, the gray module (1,830 genes; account for 25.7%) comprised the most genes, then was the turquoise one (1,295 genes; account for 18.2%), the yellow module (1,287 genes; account for 18.1%), and the brown module (1,218 genes; account for 17.1%) (Supplementary Figure 3C). The network heatmap revealed that each module was independent of the others and the genes within modules tended to show higher connectivity (Supplementary Figures 2B, 3B).

**Figure 1:**
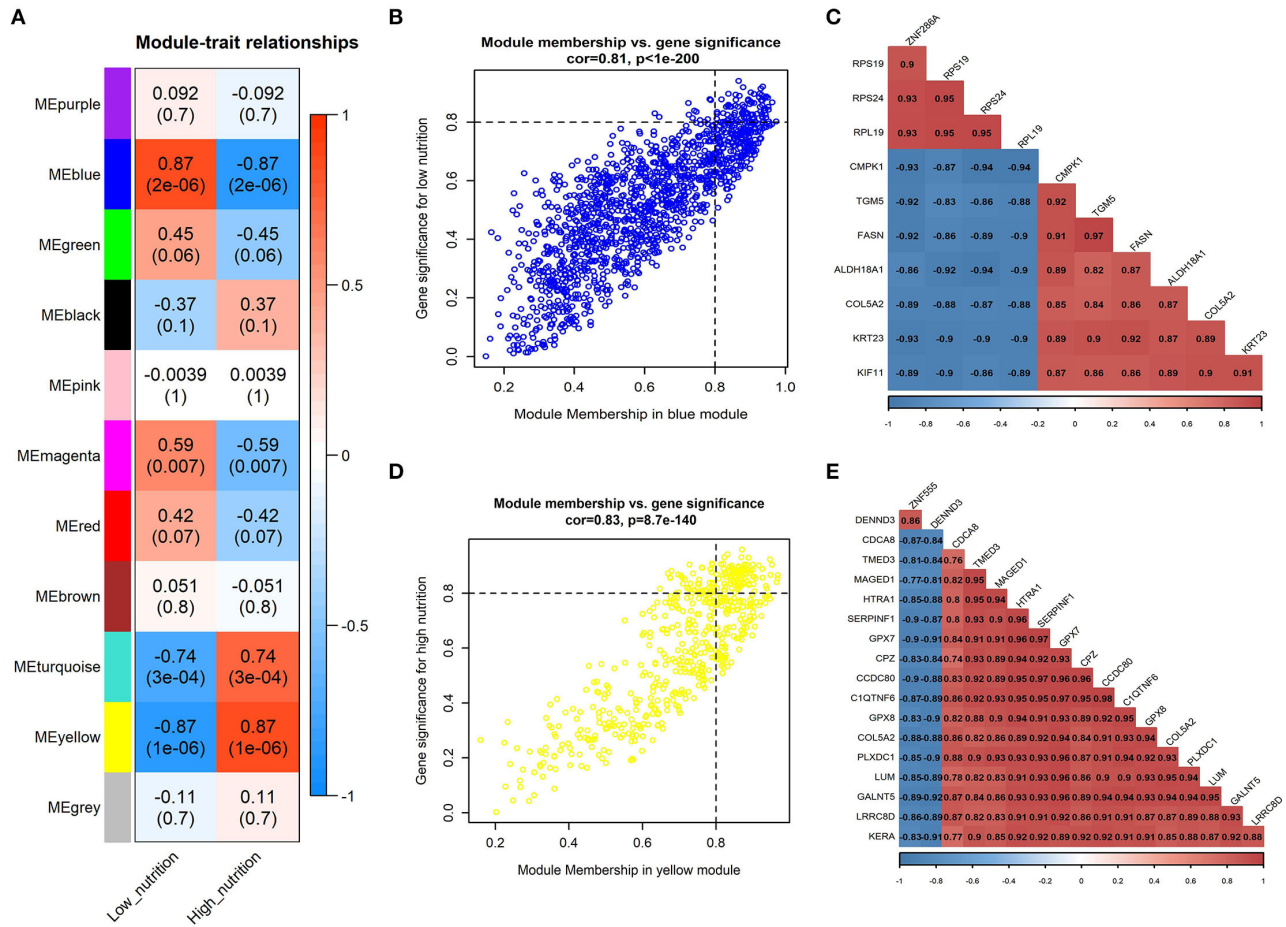
Module-trait correlations analysis in subcutaneous adipose tissue (PRJNA382633). **(A)** Correlation heatmap between modules and nutrition levels. The correlation coefficient and *P*-value are labeled in the rectangle. **(B)** The MM and GS for low nutrition in the blue module. The dots represent genes in the blue module, the horizontal axis is the MM value, and the vertical axis is the GS value for low nutrition. **(C)** The correlation relationship of the top 10 genes with high MM and GS in the blue module. The correlation coefficients are labeled in the rectangle. **(D)** The MM and GS for high nutrition in the yellow module. The dots represent genes in the yellow module, the horizontal axis is the MM value, and the vertical axis is the GS value for high nutrition. **(E)** The correlation relationship of the top 17 genes with high MM and GS in the yellow module. The correlation coefficients are labeled in the rectangle.

**Figure 2:**
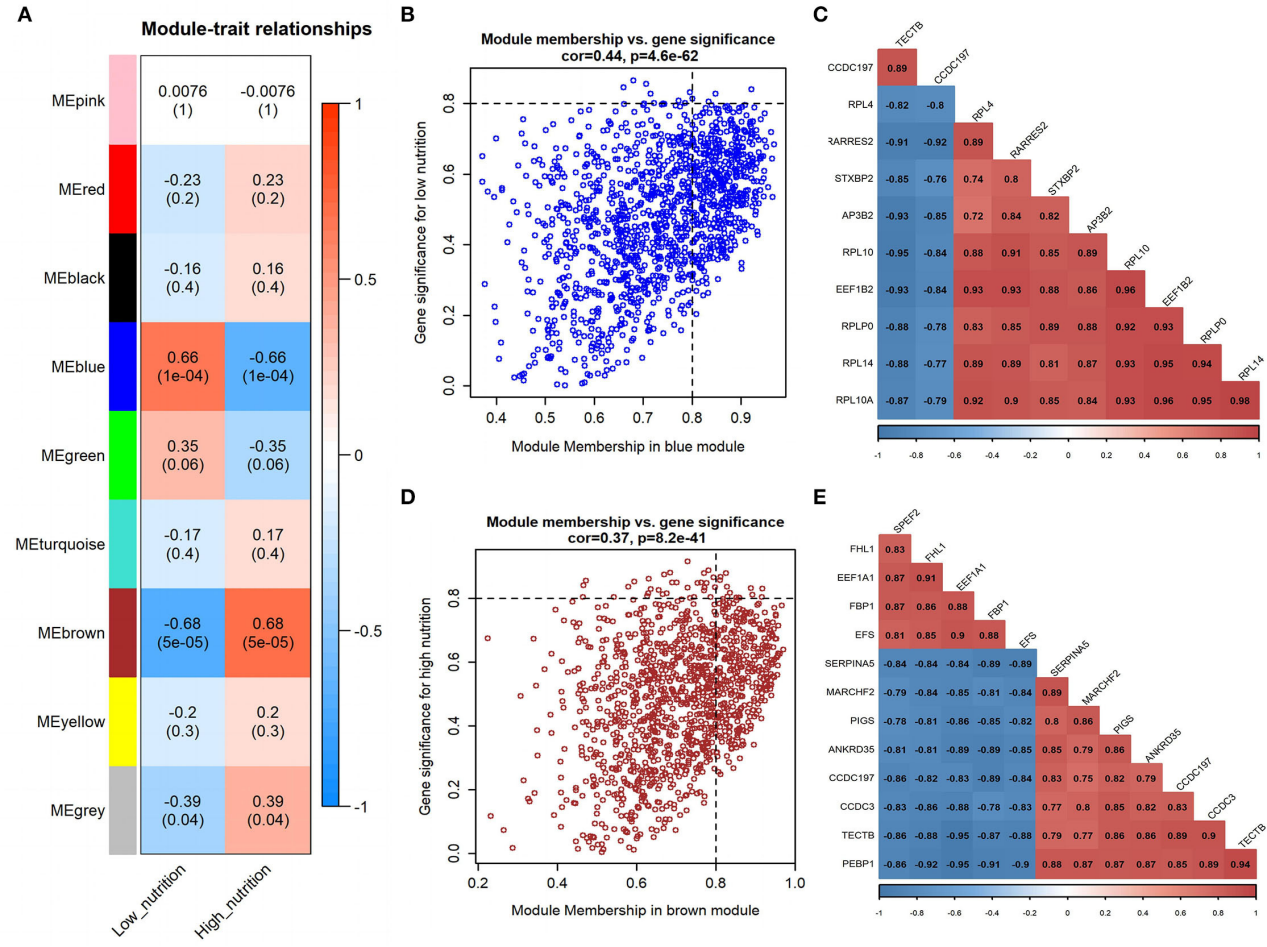
Module-trait correlations analysis in visceral adipose tissue (PRJNA664093). **(A)** Correlation heatmap between modules and nutrition levels. The correlation coefficient and *P*-value are labeled in the rectangle. **(B)** The MM and GS for low nutrition in the blue module. The dots represent genes in the blue module, the horizontal axis is the MM value, and the vertical axis is the GS value for low nutrition. **(C)** The correlation relationship of the top 10 genes with high MM and GS in the blue module. The correlation coefficients are labeled in the rectangle. **(D)** The MM and GS for high nutrition in the brown module. The dots represent genes in the brown module, the horizontal axis is the MM value, and the vertical axis is the GS value for high nutrition. **(E)** The correlation relationship of the top 12 genes with high MM and GS in the brown module. The correlation coefficients are labeled in the rectangle.

Moreover, we associated the identified modules with nutrition levels (Supplementary Figure 4). In subcutaneous adipose tissue, two modules were identified positively correlated with high and low nutrition, respectively (Figure 1A). In visceral adipose tissue, two modules positively correlated with high nutrition and one with low nutrition were identified (Figure 2A). The module with maximum correlation with the traits was selected for the subsequent analysis.

### Analysis of Modules Correlated With Low Nutrition in Subcutaneous Adipose Tissue

**Figure 3:**
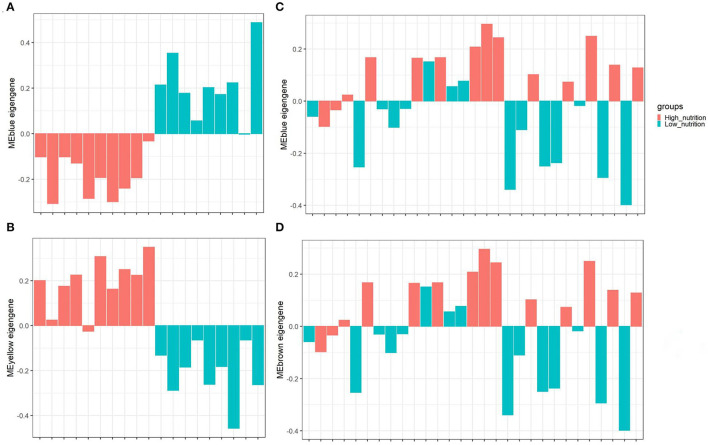
The distribution of MEs among different groups of samples. The blue module **(A)** and yellow module **(B)** identified in subcutaneous adipose tissue (PRJNA382633). The blue module **(C)** and brown module **(D)** identified in visceral adipose tissue (PRJNA664093). The horizontal axis represents the sample, and the vertical axis represents the ME value. The color represents the group of samples indicated in the upper right corner.

The result of module-trait correlation analysis revealed that the blue module showed the most significant association with low nutrition in subcutaneous adipose tissue (Figure 1A). Meanwhile, it also showed a higher ME value in the low nutrition group compared with the high (Figure 3A). The genes (such as *RPS19, RPS24*, and *RPL19*) that had high MM values and GS for low nutrition were considered potential hub genes (Figure 1B). Besides, these genes displayed a close relationship with each other (Figure 1C).

**Figure 4:**
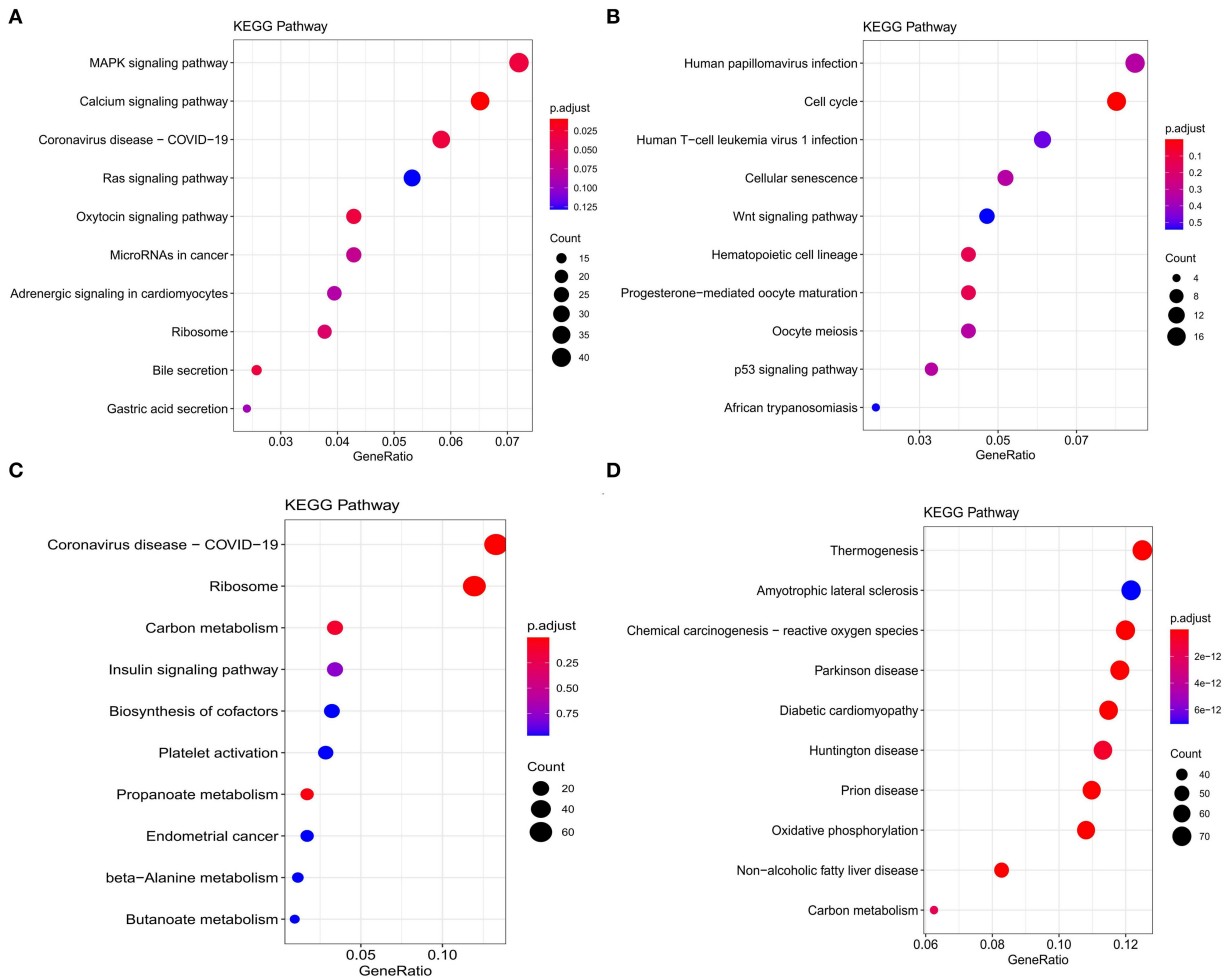
Kyoto Encyclopedia of Genes and Genomes (KEGG) functional enrichment analysis of modules that are most associated with nutrient levels. In subcutaneous adipose tissue, genes from the blue and yellow modules that significantly related to low nutrition **(A)**, and high nutrition **(B)** were analyzed. In visceral adipose tissue, genes from the blue and brown modules that significantly related to low nutrition **(C)**, and high nutrition **(D)** were analyzed. The top 10 significantly enriched pathways were shown.

**Figure 5:**
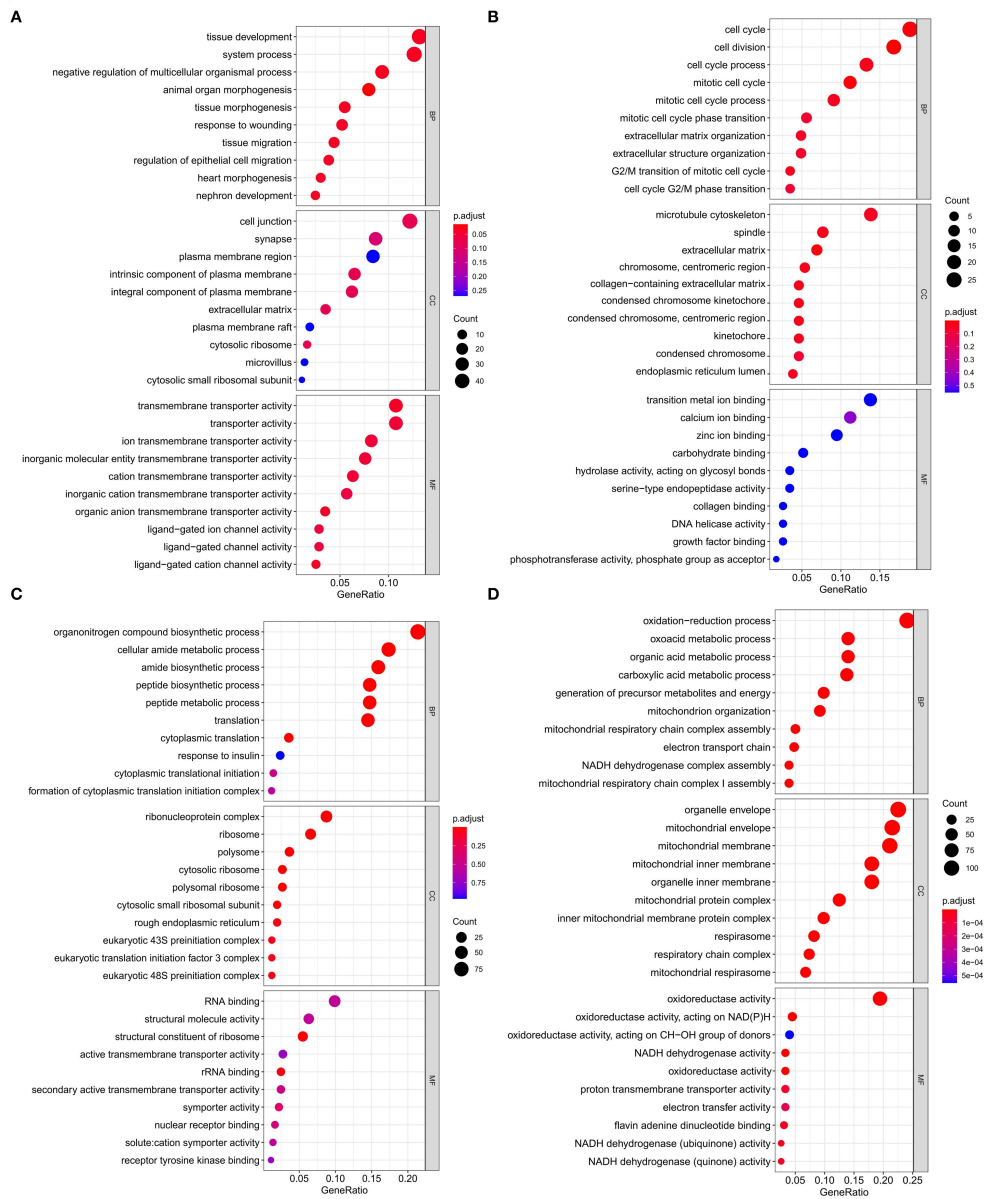
Gene ontology enrichment analysis of modules that are most associated with nutrient levels. In subcutaneous adipose tissue, genes from the blue and yellow modules that significantly related to low nutrition **(A)**, and high nutrition **(B)** were analyzed. In visceral adipose tissue, genes from the blue and brown modules that significantly related to low nutrition **(C)**, and high nutrition **(D)** were analyzed. The top 10 significant enriched GO terms were shown.

Kyoto Encyclopedia of Genes and Genomes functional enrichment analysis showed that the genes in the blue module were significantly enriched in pathways that related to fat metabolism (Ras signaling pathway and MAPK signaling pathway), reproduction (Oxytocin signaling pathway), and cell communication (Calcium signaling pathway) (Figure 4A). GO functional enrichment analysis revealed that they were mainly enriched in the biological process (BP) involved in tissue or organ development, cell components (CC) involved in the maintenance of basic cellular structure and function, and molecular function (MF) involved in multiple transmembrane transport or channel activities (Figure 5A). Therefore, we speculated that subcutaneous adipose tissue mainly played a metabolic regulation function through cell communication under a low nutrition diet.

### Analysis of Modules Correlated With High Nutrition in Subcutaneous Adipose Tissue

The yellow module showed the most significant association with high nutrition in subcutaneous adipose tissue (Figure 1A). Compared with the samples in the high nutrition group, it displayed lower ME values in the samples of low nutrition (Figure 3B). The genes (such as *DENND3, CDCA8*, and *TMED3*) that had high MM values and GS for high nutrition were considered as the potential hub genes (Figure 1D). Besides, these genes displayed a close relationship with each other (Figure 1E).

The genes in the yellow module were enriched in fat metabolism (Wnt signaling pathway), reproduction (Oocyte meiosis and Progesterone-mediated oocyte maturation), and cell proliferation and senescence (Cell cycle and Cellular senescence) pathways (Figure 4B). GO functional enrichment analysis revealed that they were mainly enriched in the BP with respect to cell cycle, CC with respect to the organization of spindle, centrosome and microtubules, and MF with respect to ion or another molecular binding (Figure 5B). In conclusion, it can be inferred that high nutrient diets might increase subcutaneous adipose tissue by promoting cell proliferation and differentiation in cattle.

### Analysis of Modules Correlated With Low Nutrition in Visceral Adipose Tissue

In visceral adipose tissue, the blue module showed the most significant association with low nutrition (Figure 2A). It also showed lower ME values in samples of low dietary nutrition compared to high nutrition (Figure 3C). The genes (such as *CCDC197, RPL4*, and *RPL10*) that had high MM values and GS for low nutrition were considered potential hub genes (Figure 2B). Besides, these genes also displayed a close relationship with each other (Figure 2C).

Genes in the blue module were significantly enriched in pathways that related to fat metabolism (insulin signaling pathway) and substance metabolism (carbon metabolism, propanoate metabolism, beta-alanine metabolism, and butanoate metabolism) (Figure 4C). GO functional enrichment analysis revealed that they were mainly enriched in the BP involved in biosynthetic and metabolic process, CC involved in ribosome organization, and MF involved in molecule binding and transporter activity (Figure 5C). Therefore, under a low nutrient diet, it can be inferred that visceral adipose tissue maintained its stability and function mainly through basal metabolism activity.

### Analysis of Modules Correlated With High Nutrition in Visceral Adipose Tissue

The brown module showed the most significant association with high nutrition in visceral adipose tissue (Figure 2A). It revealed that samples with high nutrition levels tended to have higher ME values in this module (Figure 3D). The genes (such as *FHL1, EEF1A1*, and *FBP1*) that had high MM values and GS for high nutrition were considered potential hub genes (Figure 2D). Besides, these genes are closely related to each other (Figure 2E).

Kyoto Encyclopedia of Genes and Genomes functional enrichment analysis showed that the genes in the brown module were significantly enriched in pathways that are related to energy metabolism (thermogenesis and oxidative phosphorylation) and disease (diabetic cardiomyopathy, Huntington disease, amyotrophic lateral sclerosis, etc.) (Figure 4D). GO functional enrichment analysis revealed that they were mainly enriched in the BP involved in oxidation-reduction and energy metabolism, CC involved in the mitochondrial organization, and MF involved in oxidoreductase activity (Figure 5D). It can be inferred that visceral adipose tissue may consume excess energy through mitochondrial respiration and thermogenesis to maintain a certain amount.

### Identification of Hub Genes Correlated With Low Nutrition in Subcutaneous Adipose Tissue

**Figure 6:**
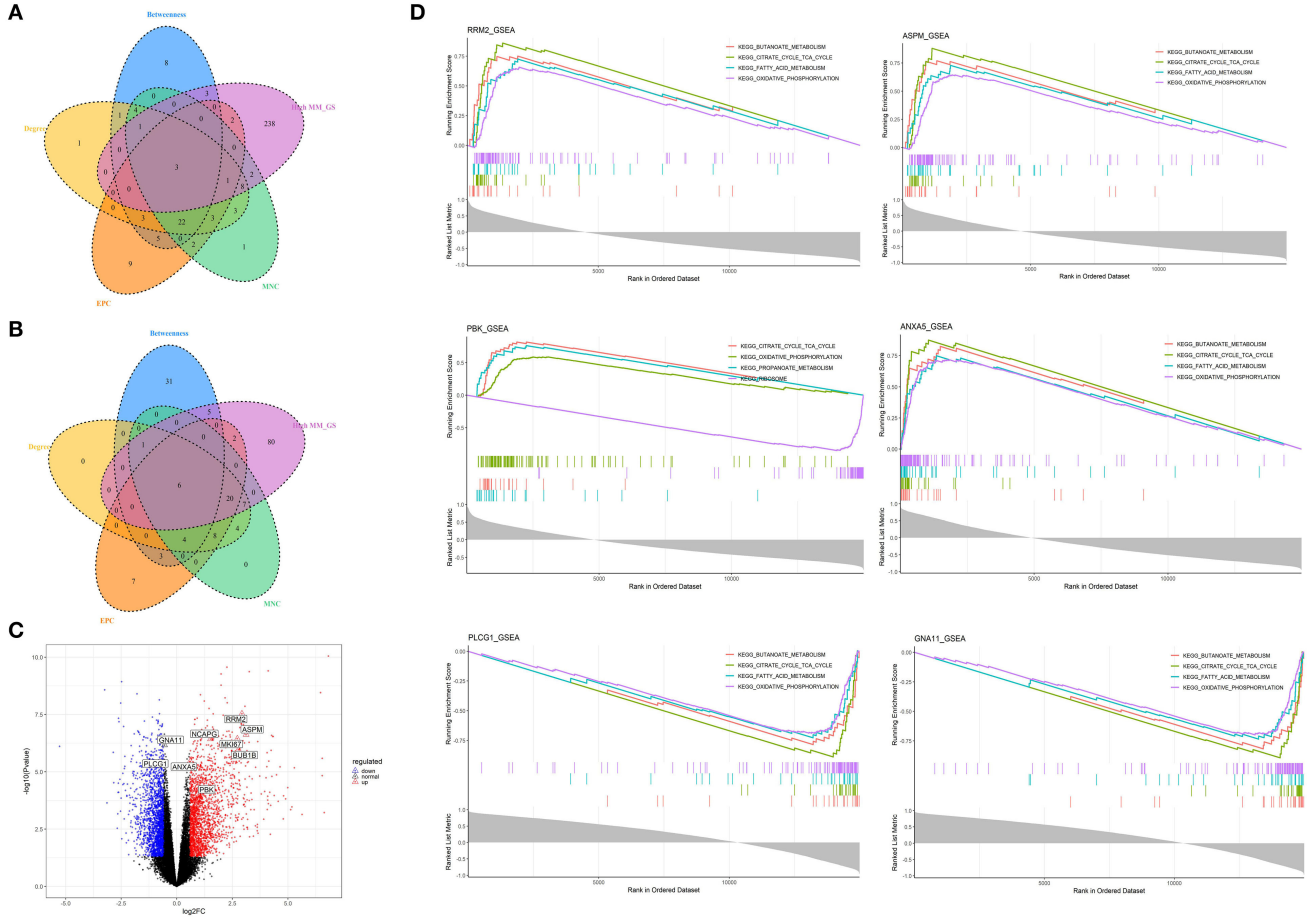
Identification and analysis of hub genes related to nutrition levels in subcutaneous adipose tissue. **(A,B)** The intersection of the potential hub genes and the top 50 hub genes identified by four algorithms (MNC, EPC, Betweenness, and Degree) in the blue module **(A)** and yellow module **(B)**. **(C)** Volcano plot of differential expressed genes between high and low nutrition levels. Red and blue dots represent the upregulated and downregulated genes comparing the high nutrition group with the low. The identified hub genes are labeled with the triangle. **(D)** GSEA analysis of hub genes related to nutrition levels in subcutaneous adipose tissue. The horizontal axis represents the ranked gene list according to the correlation with the hub gene. The vertical axis represents enrichment scores (upper) and the correlation coefficients with the hub gene (lower). Each color represents a pathway marked in the upper right corner.

We obtained 258 potential hub genes under the condition of MM >0.8 and GS >0.8 in the blue module (Figure 1B). Simultaneously, all the 1,397 genes of this module were sent to STRING to get a PPI network and the top 50 hub genes were identified by MNC, EPC, Betweenness and Degree algorithms (Supplementary Figure 5A; labeled in rhombus). *GNA11, PLCG1*, and *ANXA5* were confirmed as the final key genes by the intersection of the potential hub genes and the top 50 genes of the four algorithms (Figure 6A). Differential expression analysis showed that *PLCG1* was significantly decreased, *ANXA5* was significantly increased, while there was no significant difference with *ANXA5* (Figure 6C). Meanwhile, the Wilcoxon test revealed that the expression of *GNA11* and *PLCG1* was significantly decreased (all *P* < 0.01) in the high nutrition group compared to the low nutrition, whereas the expression of *ANXA5* was significantly increased (**Figure 8A**). Furthermore, genes negatively associated with *GNA11* and *PLCG1* were significantly enriched in pathways related to fat and energy metabolism (such as butanoate metabolism, fatty acid metabolism, citrate and TCA cycle, and oxidative phosphorylation) (Figure 6D; Supplementary Table 3).

### Identification of Hub Genes Correlated With High Nutrition in Subcutaneous Adipose Tissue

In the yellow module containing 545 genes, 121 potential hub genes were identified under the condition of MM >0.8 and GS >0.8 (Figure 1D). The top 50 hub genes were also identified by MNC, EPC, Betweenness and Degree algorithms by the PPI analysis (Supplementary Figure 5B; labeled in rhombus). Finally, six key genes (*BUB1B, ASPM, RRM2, PBK, NCAPG*, and *MKI67*) were confirmed in the high nutrition group by overlap (Figure 6B). Meanwhile, the expression of all the six hub genes showed a significant increase in the high nutrition group compared to the low nutrition (Figures 6C, **8B**). Furthermore, genes positively associated with the six hub genes were significantly enriched in the pathways related to fat and energy metabolism (Figure 6D; Supplementary Table 3).

### Identification of Hub Genes Correlated With Low Nutrition in Visceral Adipose Tissue

**Figure 7:**
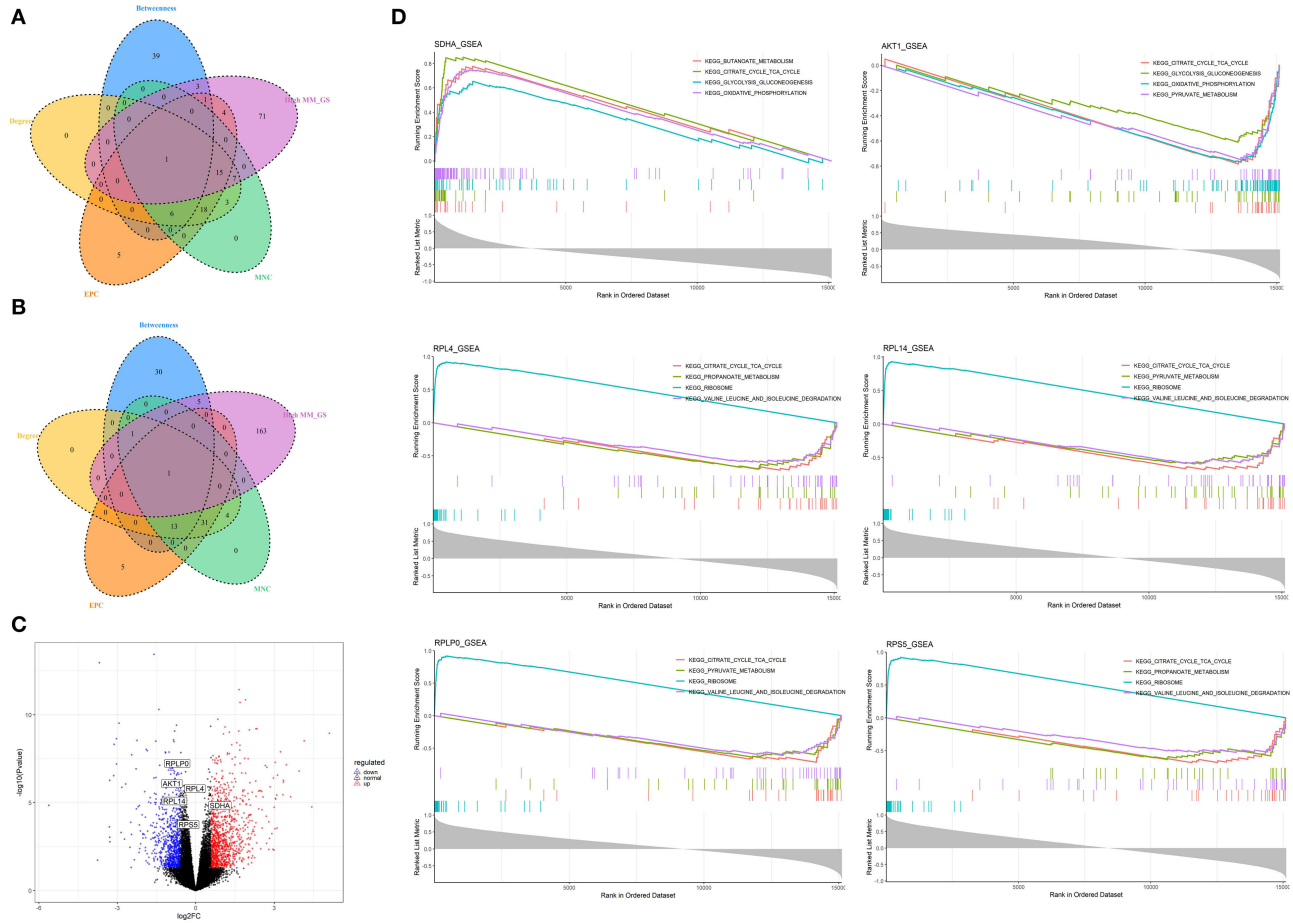
Identification and analysis of hub genes related to nutrition levels in visceral adipose tissue. **(A,B)** The intersection of the potential hub genes and the top 50 hub genes identified by four algorithms (MNC, EPC, Betweenness, and Degree) in the blue module **(A)** and brown module **(B)**. **(C)** Volcano plot of differential expressed genes between high and low nutrition levels. Red and blue dots represent the upregulated and downregulated genes comparing the high nutrition group with the low. The identified hub genes are labeled with the triangle. **(D)** GSEA analysis of hub genes related to nutrition levels in visceral adipose tissue. The horizontal axis represents the ranked gene list according to the correlation with the hub gene. The vertical axis represents enrichment scores (upper) and the correlation coefficients with the hub gene (lower). Each color represents a pathway marked in the upper right corner.

**Figure 8:**
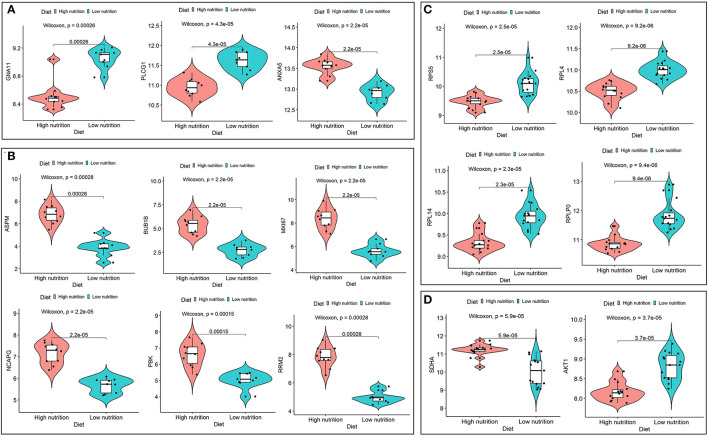
Expression analysis of hub genes under high and low levels of nutrition. Expression levels of hub genes for low nutrition **(A)**, and high nutrition **(B)** in subcutaneous adipose tissue, and hub genes for low nutrition **(C)**, and high nutrition **(D)** in visceral adipose tissue.

A total of 102 potential hub genes were obtained with MM >0.7 and GS >0.8 in the blue module (Figure 2B). Simultaneously, all the 1,287 genes of this module were used to construct a PPI network. Only one gene, *RPS5*, was obtained by the intersection of the potential hub genes and the top 50 genes of the above four algorithms (Supplementary Figure 5). Figure 7A showed that 15 genes can be obtained excluding the algorithm Betweenness. Furthermore, the other three core genes (*RPL4, RPL14*, and *RPLP0*) were obtained by the intersection of these 15 genes with the top 10 genes with MM and GS values (Supplementary Figure 5C; labeled in rhombus). Although the differential expression of *RPL4, RPL14*, and *RPS5* was not significant (Figure 7C), the Wilcoxon test showed that the four final hub genes were all significantly decreased (*P* < 0.01) in the high nutrition group compared to the low nutrition (Figure 8C). Furthermore, genes positively associated with the four final hub genes were significantly enriched in pathways related to the ribosome (Figure 7D). The genes negatively associated with them were significantly enriched in pathways related to energy and substance metabolism (such as citrate and TCA cycle, propanoate metabolism, and valine, leucine, and isoleucine degradation) (Figure 7D; Supplementary Table 4).

### Identification of Hub Genes Correlated With High Nutrition in Visceral Adipose Tissue

When the criteria were set as MM >0.7 and GS >0.7, 169 potential hub genes were obtained in the brown module (Figure 2D). Simultaneously, all the 1,218 genes of this module were sent to STRING to get a PPI network and *SDHA* was confirmed as the hub gene by the overlap of the top 50 of the four algorithms (Supplementary Figure 5D; labeled in rhombus). Besides, there is another gene (*AKT1*) identified by the intersection excluding the algorithm EPC (Figure 7B). Finally, *SDHA* and *AKT1* were confirmed as the key genes. The expression of *SDHA* was significantly increased in the high nutrition group compared to the low nutrition, whereas the expression of *AKT1* was significantly decreased (Figures 7C, 8D). Furthermore, genes positively associated with *SDHA* and negatively associated with *AKT1* were significantly enriched in pathways such as citrate and TCA cycle, oxidative phosphorylation, and glycolysis and gluconeogenesis (Figure 7D; Supplementary Table 4).

### Evaluation of Hub Genes by ROC Curve

**Figure 9:**
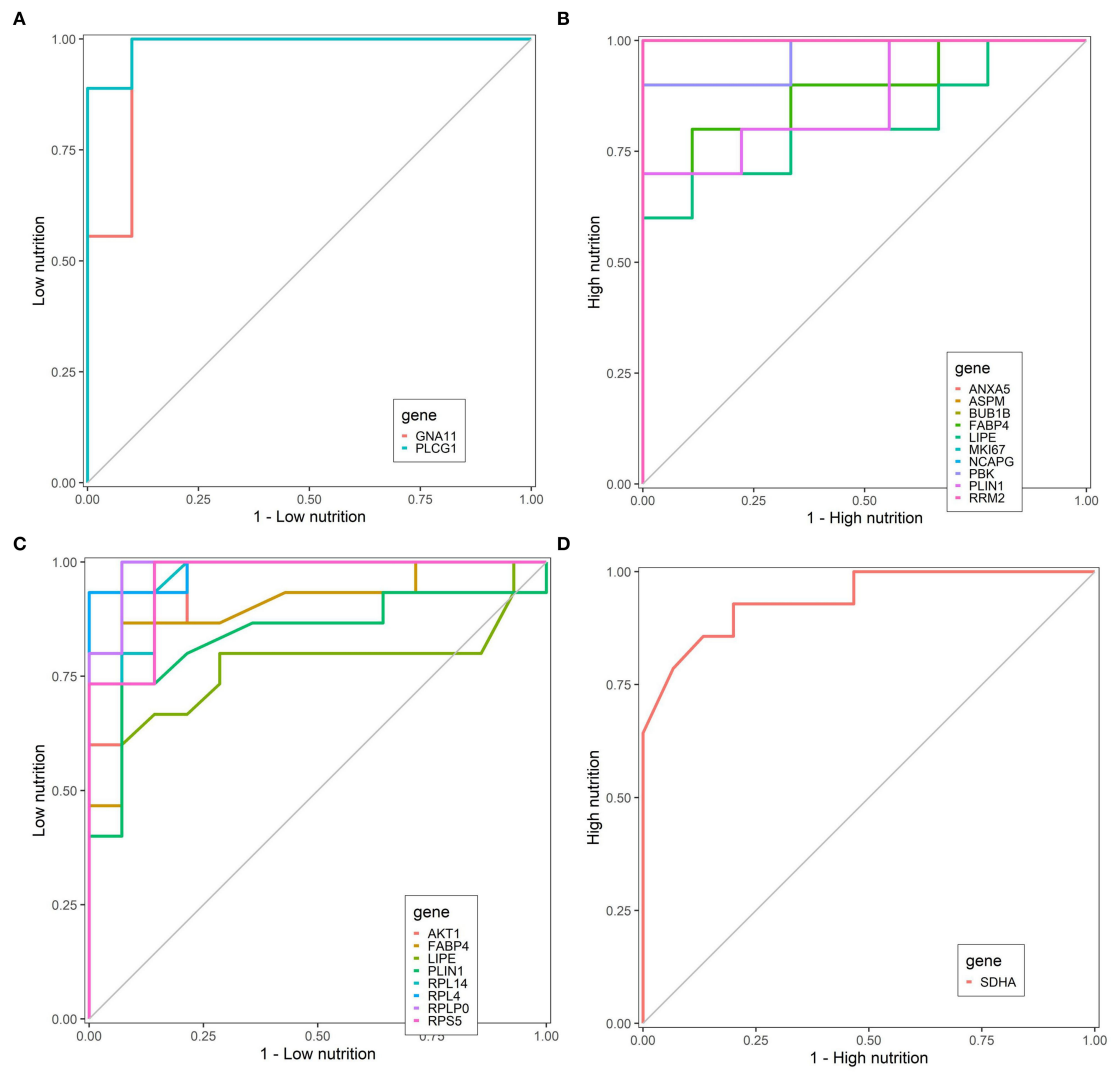
Receiver operating characteristic (ROC) curve analysis of hub genes. Hub genes can distinguish low nutrition **(A)** and high nutrition **(B)** in subcutaneous adipose tissue, and hub genes can distinguish low nutrition **(C)** and high nutrition **(D)** in visceral adipose tissue. Genes are represented by different colored lines. The larger area under the curve indicates that the genes have a stronger ability to differentiate the high and low nutrition groups.

Finally, we identified nine core genes in subcutaneous adipose tissue, three of which were significantly associated with a low nutrient diet and six with a high nutrient diet (Table 1). Meanwhile, six core genes were obtained in visceral adipose tissue, four of which were significantly associated with a low nutrient diet and two with a high nutrient diet (Table 1). ROC curve was plotted and the area under the curve (AUC) was calculated to distinguish high and low nutrient diet groups. In subcutaneous adipose tissue, *GNA11* and *PLCG1* can distinguish a low nutrient diet from a high nutrient diet (AUC > 0.7) (Figure 9A), and *ANXA5, BUB1B, ASPM, RRM2, PBK, NCAPG*, and *MKI67* can distinguish high nutrient diet from the low nutrient diet (AUC > 0.7) (Figure 9B). In visceral adipose tissue, *RPS5, RPL4, RPL14, RPLP0*, and *AKT1* have the ability to distinguish a low nutrient diet from a high nutrient diet (AUC > 0.7) (Figure 9C), and *SDHA* has the ability to distinguish high nutrient diet from the low nutrient diet (AUC > 0.7) (Figure 9D).

**Table 1:** The hub genes identified in subcutaneous and visceral adipose tissues.

**Fat types**	**Diet**	**Symbol**	**Description**	**Direct function**
Subcutaneous adipose tissue	Low plane of nutrition	PLCG1	Phospholipase C gamma 1	Catalyzes the formation of inositol 1,4,5-trisphosphate and diacylglycerol
		GNA11	G protein subunit alpha 11	Modulators or transducers in various transmembrane signaling systems
		ANXA5	Annexin A5	Calcium channel activity; cellular signal transduction, inflammation, growth and differentiation
	High plane of nutrition	BUB1B	BUB1 mitotic checkpoint serine/threonine kinase B	A kinase involved in spindle checkpoint function
		ASPM	Assembly factor for spindle microtubules	Mitotic spindle regulation
		RRM2	Ribonucleotide reductase regulatory subunit M2	Catalyzes the formation of deoxyribonucleotides from ribonucleotides
		PBK	PDZ binding kinase	Mitotic phosphorylation
		NCAPG	Non-SMC condensin I complex subunit G	Condensation and stabilization of chromosomes during mitosis and meiosis
		MKI67	Marker of proliferation Ki-67	A nuclear protein that is associated with cellular proliferation
Visceral adipose tissues	Low plane of nutrition	RPS5	Ribosomal protein S5	Participation in ribosome organization and catalysis of protein synthesis
		RPL4	Ribosomal protein L4	
		RPL14	Ribosomal protein L14	
		RPLP0	Ribosomal protein lateral stalk subunit P0	
	High plane of nutrition	SDHA	Succinate dehydrogenase complex flavoprotein subunit A	Mitochondrial respiratory chain
		AKT1	AKT serine/threonine kinase 1	Binding of membrane-bound ligands

## Discussion

Subcutaneous adipose tissue is mainly distributed below the dermis and above the fascia layer, and is generally considered benign fat for its significant function in energy storage and utilization, protection, and cold resistance (28). Visceral fat is generally located around the visceral organs in the abdominal cavity and is generally considered malignant fat for its association with the occurrence of a variety of diseases (29). Meanwhile, subcutaneous and visceral fat, as two important adipose tissue types, could synthesize and secrete adipokines to play a variety of metabolic regulation functions. However, they differed in gene expression and regulatory mechanisms due to the heterogeneity of adipose tissue (30, 31). Previous studies have shown that a high plane of nutrition led to the differential accumulation of adipose tissue in various depots, such as subcutaneous, intramuscular, visceral, and epididymal (32). Excess energy tended to be converted to subcutaneous fat first, followed by visceral and intramuscular fat. It has also been proved that there are significant differences in phenotypic structure and lipid metabolism between subcutaneous and visceral adipose tissue (33). Therefore, it is inferred that these two kinds of adipose tissues might exhibit various regulatory mechanisms under different nutrition levels. The purpose of this study was to explore the expression networks in subcutaneous and visceral adipose tissues under high and low dietary nutrition conditions in early calf-hood, and to obtain key genes related to nutrition levels, thus providing a reference for bovine breeding.

The site of fat deposition plays a significant role in meat quality and animal health (33). Subcutaneous fat mainly affects the economic value through backfat thickness and carcass weight in cattle and pigs (34–36). Besides, the subcutaneous depots protect systemic glucose homeostasis to maintain normal metabolic activity, and removal of them potentially leads to glucose intolerance because of the decreased storage space for glucose and/or lipids (37). In this study, functional enrichment analysis revealed that subcutaneous adipose tissue played a regulatory function mainly by secreting cytokines under the condition of a low nutrition diet, while excess energy was converted into fat depots through cell proliferation and differentiation under the condition of a high nutrition diet (Supplementary Table 5). The enrichment results of the interested modules were also consistent with the results of GSEA and GSVA analysis (Supplementary Figures 6, 7; Supplementary Table 5).

Furthermore, the three hub genes (*PLCG1, GNA11*, and *ANXA5*) associated with a low nutrient diet are mainly involved in KEGG pathways that are related to fat metabolism and signal transduction (Supplementary Figure 8). The protein encoded by *PLCG1* catalyzes the formation of inositol 1,4,5-trisphosphate and diacylglycerol from phosphatidylinositol 4,5-bisphosphate (38). In this process, calcium was used as a co-factor and functions in the intracellular transduction of receptor-mediated tyrosine kinase activators. The activated PLCG1 by SRC caused the translocation of Ras guanine nucleotide exchange factor (RasGRP1) to Golgi to activate Ras (39). Meanwhile, it has been proved that the Ras and Ras signaling pathway regulate glucose uptake and fat metabolism in a diet-dependent manner (40, 41).

The proteins encoded *GNA11* and *ANXA5* function as modulators or transducers in various transmembrane signaling systems (Table 1) (42–44). Pathway analysis revealed that *GNA11* participates in *the* GnRH signaling pathway and Growth hormone synthesis, secretion, and action (Supplementary Figure 8), which played an important role in the regulation of reproductive physiology (45). A previous study that used the same dataset has shown that the high plane of nutrition caused up-regulation of reproduction-related genes, such as leptin (*LEP*) and adiponectin (*ADIPOQ*) (9). *GNA11*, as a newly identified key gene associated with reproduction, complements the results of previous studies. By means of functional enrichment analysis and consulting literature, it was found that the six hub genes associated with a high nutrient diet are mainly involved in the cell cycle and mitosis (Table 1; Supplementary Figure 8). In addition, the marker genes of fat metabolism showed significantly higher expression in the high nutrition diet group compared with the low nutrition diet group (Supplementary Figure 9). These genes may function in converting excess energy into lipid and storing it in adipose tissue by promoting cell proliferation and differentiation.

In the dairy and beef cattle industry, it is important to produce high-quality products at a low cost. Visceral adipose tissue, as an endocrine organ, could secrete several cytokines to regulate the development of systemic inflammation, insulin resistance, and related diseases (28). Abnormally deposition of visceral adipose tissue indicated a higher risk of metabolic disorders and disease (46). Therefore, studying the effect of dietary nutrition levels on visceral fat is beneficial to promoting healthy breeding, reducing the occurrence of disease, and improving economic income in the cattle industry. We obtained six genes associated with nutrition levels in visceral adipose tissue, of which four were associated with a low nutrient diet and two with a high nutrient diet (Table 1; Supplementary Figure 5). The four key genes associated with a low nutrition diet mainly regulated the synthesis of peptides and proteins through participation in ribosome organization (Table 1). The peptides and proteins that act as regulatory molecules were secreted and transported throughout the body to elicit and control a wide variety of biological actions (47).

The two key genes (*SDHA* and *AKT1*) associated with high nutrition diet mainly play a role in consuming excess energy through mitochondrial respiration and thermogenesis (Table 1; Supplementary Figure 8). Previous studies have also shown that they were differentially expressed genes between high and low nutrition diet groups, and participate in the Sirtuin signaling pathway that involved in energy metabolism (10). As a milk protein related to mitochondrial oxidative metabolism, SDHA was proposed as a putative biomarker of negative energy balance based on its implication in metabolic adaptative pathways (48). As an important component of the mitochondrial respiratory chain, the hepatic *SDHA* expression of low residual feed intake (RFI) steers was significantly higher than that of high RFI steers (49). This can be attributed to greater hepatic mitochondrial density and function that lead to the higher efficiency in hepatic nutrient metabolism, which has a positive correlation with feed efficiency (low-RFI). Proteome sequencing of bovine subcutaneous adipose tissue showed that *SDHA* was one of the differentially expressed genes between 12 and 15 months involved in lipid metabolism (50). The other hub gene, *AKT1*, is a key component of AKT/PI3K signaling pathways, which regulate a wide variety of cellular functions including cell proliferation, survival, metabolism, and angiogenesis. For example, *AKT1* could regulate bovine granulosa cell apoptosis *via* PI3K/AKT and ERK1/2 signaling pathways to affect the reproductive performance of cows (51). The previous study also revealed that enhanced nutrition during early calfhood facilitates reproductive performance by regulating the hormones in the hypothalamic-pituitary-ovarian (HPO) axis (23). In addition, compared with subcutaneous fat, nutrition levels had less effect on the expression of marker genes related to fat metabolism in visceral fat (Supplementary Figure 9). This may also explain that visceral fat is a potential endocrine organ rather than energy storage.

It is of great significance for bovine breeding to obtain core genes related to nutrition levels by analyzing the molecular regulatory network of subcutaneous and visceral adipose tissue. As far as we know, our study is the first one to build and analyze the nutrition-related gene network in the two adipose tissue types. Several gene co-expression modules and hub genes associated with a high and low plane of nutrition were identified, presenting insights into the function and regulatory mechanism of early calf-hood nutrition on visceral and subcutaneous adipose tissues. There are also limitations in our research. For instance, the adipose tissue samples were derived from two datasets, providing calves with very similar but not identical dietary regimes. This could potentially affect the results. In addition, we only obtained the key genes through analysis but did not carry out further experimental verification.

## Conclusions

In summary, understanding the mechanism of metabolism differences related to nutrition levels could help to improve the production efficiency in the cattle industry. In this study, gene co-expression analysis was conducted in subcutaneous and visceral adipose tissues of calves feeding on low or high dietary nutrition for more than 100 days and found the involvement of the co-expression modules and functional biological pathways related to nutrition levels. Meanwhile, key genes in subcutaneous adipose tissue for low nutrition (*PLCG1, GNA11*, and *ANXA5*) and high nutrition (*BUB1B, ASPM, RRM2, PBK, NCAPG*, and *MKI67*), and visceral adipose tissue for low nutrition (*RPS5, RPL4, RPL14*, and *RPLP0*) and high nutrition (*SDHA* and *AKT1*) were obtained and verified. These findings provide new insights into the effect of early calf-hood nutrition on the two adipose tissues and the hub genes identified could be candidates to further explore their exact molecular mechanism.

## Data Availability Statement

The original contributions presented in the study are included in the article/Supplementary Material, further inquiries can be directed to the corresponding author/s.

## Author Contributions

YuM and CP: conceived and designed the research. CP and CY: analyzed the data. CP: wrote the manuscript. YuM, CY, YaM, HS, ZL, SW, HH, XF, and JZ: modified the manuscript. All authors read and approved the final manuscript.

## Funding

This study was funded by the National Natural Science Foundation of China (32072720), the Key Research and Talent Introduction Project of Ningxia Hui Autonomous Region (2021BEF01002 and 2021NXZD1), the Leading Talents Fund in Science and Technology Innovation in Ningxia Hui Autonomous Region (2020GKLRLX02), the Modern Agroindustry Technology Research System (CARS-36), and the Development and Application of Health Control Technology for High Yield Dairy Cows (No. 2021BEF01001). The funding bodies played no role in the design of the study, collection, analysis, and interpretation of data and writing the manuscript.

## Conflict of Interest

The authors declare that the research was conducted in the absence of any commercial or financial relationships that could be construed as a potential conflict of interest.

## Publisher's Note

All claims expressed in this article are solely those of the authors and do not necessarily represent those of their affiliated organizations, or those of the publisher, the editors and the reviewers. Any product that may be evaluated in this article, or claim that may be made by its manufacturer, is not guaranteed or endorsed by the publisher.

## Supplementary Material

Supplementary Figure 1 Sample dendrogram and trait heatmap in PRJNA382633 (left) and PRJNA664093 (right).

Supplementary Figure 2 Gene co-expression networks in subcutaneous adipose tissue (PRJNA382633). **(A)** Analysis of the scale-free fit index for soft-thresholding powers (left) and the mean connectivity for various soft-thresholding powers (right); **(B)** Network heatmap plot in the co-expression modules. **(C)** Gene clustering dendrogram and module identification.

Supplementary Figure 3 Gene co-expression networks in visceral adipose tissue (PRJNA664093). **(A)** Analysis of the scale-free fit index for soft-thresholding powers (left) and the mean connectivity for various soft-thresholding powers (right); **(B)** Network heatmap plot in the co-expression modules. **(C)** Gene clustering dendrogram and module identification.

Supplementary Figure 4 The relationships among the module eigengenes and nutritional levels in subcutaneous adipose tissue (left) and visceral adipose tissue (right).

Supplementary Figure 5 Top 50 hub genes identified by Betweenness, Degree, EPC, and MNC in the blue **(A)** and yellow **(B)** module of dataset PRJNA382633 (subcutaneous adipose tissue), and blue **(C)** and brown **(D)** module of dataset PRJNA664093 (visceral adipose tissue).

Supplementary Figure 6 Gene set variation analysis (GSVA) results in subcutaneous **(A)** and visceral **(B)** adipose tissues.

Supplementary Figure 7 Gene set enrichment analysis (GSEA) results in subcutaneous adipose tissue **(A)**, and visceral adipose tissue **(B)**. The horizontal axis represents the ranked gene list according to the log2 FC values between high and low nutrition groups. The vertical axis represents enrichment scores (upper) and the log2 FC values (lower). Each color represents a pathway marked in the upper right corner.

Supplementary Figure 8 Circos plot to indicate the relationship between hub genes and KEGG pathways.

Supplementary Figure 9 Expression analysis of marker genes of fat metabolism in subcutaneous **(A)** and visceral **(B)** adipose tissues.

Supplementary Table 1 Experimental and phenotypical information of the used datasets (PRJNA382633 and PRJNA664093).

Supplementary Table 2 Modules and genes identified in subcutaneous adipose tissue (PRJNA382633) and visceral adipose tissue (PRJNA664093).

Supplementary Table 3 Single-gene gene set enrichment analysis (GSEA) results in hub genes in subcutaneous adipose tissue.

Supplementary Table 4 Single-gene GSEA results in hub genes in visceral adipose tissue.

Supplementary Table 5 Gene set enrichment analysis results in subcutaneous and visceral adipose tissues.

## References

[1] Zhang S Zhong G Shao D Wang Q Hu Y Wu T. Dietary supplementation with Bacillus subtilis promotes growth performance of broilers by altering the dominant microbial community. Science. (2021) 100:100935. 10.1016/j.psj.2020.12.032 33652528 PMC7936199

[2] Havekes CD Duffield TF Carpenter AJ DeVries TJ. Moisture content of high-straw dry cow diets affects intake, health, and performance of transition dairy cows. J Dairy Sci. (2020) 103:1500–15. 10.3168/jds.2019-17557 31837778

[3] Xiao Y Zhang S Tong H Shi S. Comprehensive evaluation of the role of soy and isoflavone supplementation in humans and animals over the past two decades. Phytother Res. (2018) 32:384–94. 10.1002/ptr.5966 29193539

[4] Havekes CD Duffield TF Carpenter AJ DeVries TJ. Effects of molasses-based liquid feed supplementation to a high-straw dry cow diet on feed intake, health, and performance of dairy cows across the transition period. J Dairy Sci. (2020) 103:5070–89. 10.3168/jds.2019-18085 32278564

[5] Qiu Q Qiu X Gao C Muhammad A Cao B Su H. High-density diet improves growth performance and beef yield but affects negatively on serum metabolism and visceral morphology of Holstein steers. J Anim Physiol Anim Nutr. (2020) 104:1197–208. 10.1111/jpn.13340 32190937

[6] Formigoni A Trevisi E. Transition cow: interaction with fertility. Vet Res Commun. (2003) 27(Suppl. 1):143–52. 10.1023/b:verc.0000014131.34839.4c 14535382

[7] Rodney RM Celi P Scott W Breinhild K Lean IJ. Effects of dietary fat on fertility of dairy cattle: a meta-analysis and meta-regression. J Dairy Sci. (2015) 98:5601–20. 10.3168/jds.2015-9528 26094218

[8] Harlow K Taylor E Casey T Hedrick V Sobreira T Aryal UK. Diet impacts pre-implantation histotroph proteomes in beef cattle. J Proteome Res. (2018) 17:2144–55. 10.1021/acs.jproteome.8b00077 29722258

[9] English A Waters SM Cormican P Byrne CJ Fair S Kenny DA. (2018). Effect of early calf-hood nutrition on the transcriptomic profile of subcutaneous adipose tissue in Holstein-Friesian bulls. BMC Genomics. 19:281. 10.1186/s12864-018-4681-2 PMC5916831 29690861

[10] Keogh K Kelly AK Kenny DA. Effect of plane of nutrition in early life on the transcriptome of visceral adipose tissue in Angus heifer calves. Sci Rep. (2021) 11:9716. 10.1038/s41598-021-89252-x 33958675 PMC8102595

[11] Smitka K Maresova D. Adipose tissue as an endocrine organ: an update on pro-inflammatory and anti-inflammatory microenvironment. Prague Med Rep. (2015) 116:87–111. 10.14712/23362936.2015.49 26093665

[12] Shen Y Zhang S Zhao X Shi S. Evaluation of a lecithin supplementation on growth performance, meat quality, lipid metabolism, and cecum microbiota of broilers. Animals. (2021) 11:2537. 10.3390/ani11092537 34573503 PMC8465824

[13] Ouchi N Parker JL Lugus JJ Walsh K. Adipokines in inflammation and metabolic disease. Nat Rev Immunol. (2011) 11:85–97. 10.1038/nri2921 21252989 PMC3518031

[14] Shingfield KJ Bernard L Leroux C Chilliard Y. Role of trans fatty acids in the nutritional regulation of mammary lipogenesis in ruminants. Animal. (2010) 4:1140–66. 10.1017/S1751731110000510 22444614

[15] Lopreiato V Hosseini A Rosa F Zhou Z Alharthi A Trevisi E. Dietary energy level affects adipose depot mass but does not impair *in vitro* subcutaneous adipose tissue response to short-term insulin and tumor necrosis factor-α challenge in nonlactating, nonpregnant Holstein cows. J Dairy Sci. (2018) 101:10206–19. 10.3168/jds.2018-14389 30146294

[16] Drackley JK Wallace RL Graugnard D Vasquez J Richards BF Loor JJ. Visceral adipose tissue mass in nonlactating dairy cows fed diets differing in energy density(1). J Dairy Sci. (2014) 97:3420–30. 10.3168/jds.2014-8014 24704224

[17] Weber M Locher L Huber K Kenez A Rehage J Tienken R. Longitudinal changes in adipose tissue of dairy cows from late pregnancy to lactation. Part 1: the adipokines apelin and resistin and their relationship to receptors linked with lipolysis. J Dairy Sci. (2016) 99:1549–59. 10.3168/jds.2015-10131 26686707

[18] Langfelder P Horvath S WGCNA. An R package for weighted correlation network analysis. BMC Bioinformatics. (2008) 9:559. 10.1186/1471-2105-9-559 19114008 PMC2631488

[19] Riquelme MI Lubovac-Pilav Z. Gene Co-Expression network analysis for identifying modules and functionally enriched pathways in type 1 diabetes. PLoS ONE. (2016) 11:e156006. 10.1371/journal.pone.0156006 27257970 PMC4892488

[20] Yao Q Song Z Wang B Qin Q Zhang J. Identifying key genes and functionally enriched pathways in sjögren's syndrome by weighted gene co-expression network analysis. Front Genet. (2019) 10:1142. 10.3389/fgene.2019.01142 31798636 PMC6863930

[21] Ma C Lv Q Teng S Yu Y Niu K Yi C. Identifying key genes in rheumatoid arthritis by weighted gene co-expression network analysis. Int J Rheum Dis. (2017) 20:971–9. 10.1111/1756-185X.13063 28440025

[22] Cai Y Ma F Qu L Liu B Xiong H Ma Y. Weighted gene co-expression network analysis of key biomarkers associated with bronchopulmonary dysplasia. Front Genet. (2020) 11:539292. 10.3389/fgene.2020.539292 33033495 PMC7509191

[23] Kelly AK Byrne C McGee M Perry GA Crowe MA Sauerwein H. Effect of calfhood nutrition on metabolic hormones, gonadotropins, and estradiol concentrations and on reproductive organ development in beef heifer calves. J Anim Sci. (2020) 98:skaa310. 10.1093/jas/skaa310 32954407 PMC7603402

[24] Szklarczyk D Gable AL Nastou KC Lyon D Kirsch R Pyysalo S. The STRING database in 2021: Customizable protein-protein networks, and functional characterization of user-uploaded gene/measurement sets. Nucleic Acids Res. (2021) 49:D605–12. 10.1093/nar/gkaa1074 33237311 PMC7779004

[25] Doncheva NT Morris JH Gorodkin J Jensen LJ. Cytoscape StringApp: network analysis and visualization of proteomics data. J Proteome Res. (2019) 18:623–32. 10.1021/acs.jproteome.8b00702 30450911 PMC6800166

[26] Yu G Wang LG Han Y He QY. ClusterProfiler: an R package for comparing biological themes among gene clusters. OMICS. (2012) 16:284–7. 10.1089/omi.2011.0118 22455463 PMC3339379

[27] Robin X Turck N Hainard A Tiberti N Lisacek F Sanchez J. PROC: An open-source package for R and S+ to analyze and compare ROC curves. BMC Bioinformatics. (2011) 12:77. 10.1186/1471-2105-12-77 21414208 PMC3068975

[28] Ibrahim MM. Subcutaneous and visceral adipose tissue: structural and functional differences. Obes Rev. (2010) 11:11–8. 10.1111/j.1467-789X.2009.00623.x 19656312

[29] Tchernof A Despres JP. Pathophysiology of human visceral obesity: an update. Physiol Rev. (2013) 93:359–404. 10.1152/physrev.00033.2011 23303913

[30] Schweizer R Waldner M Oksuz S Zhang W Komatsu C Plock JA. Evaluation of porcine versus human mesenchymal stromal cells from three distinct donor locations for cytotherapy. Front Immunol. (2020) 11:826. 10.3389/fimmu.2020.00826 32435248 PMC7218165

[31] Silva KR Baptista LS. Adipose-derived stromal/stem cells from different adipose depots in obesity development. World J Stem Cells. (2019) 11:147–66. 10.4252/wjsc.v11.i3.147 30949294 PMC6441940

[32] Lee MJ Wu Y Fried SK. Adipose tissue heterogeneity: implication of depot differences in adipose tissue for obesity complications. Mol Aspects Med. (2013) 34:1–11. 10.1016/j.mam.2012.10.001 23068073 PMC3549425

[33] Wang S Liu J Zhao W Wang G Gao S. Selection of candidate genes for differences in fat metabolism between cattle subcutaneous and perirenal adipose tissue based on RNA-seq. Anim Biotechnol. (2021) 23:1–12. 10.1080/10495398.2021.1991937 34693889

[34] Davoli R Catillo G Serra A Zappaterra M Zambonelli P Zilio DM. Genetic parameters of backfat fatty acids and carcass traits in Large White pigs. Animal. (2019) 13:924–32. 10.1017/S1751731118002082 30152309

[35] Pinheiro TR Mercadante ME Albuquerque LG Cyrillo JN Branco RH. Phenotypic and genetic parameters compared during repeated measures of longissimus muscle area and subcutaneous fat thickness in Nelore cattle. Genet Mol Res. (2011) 10:2944–52. 10.4238/2011.November.29.5 22179966

[36] Zambonelli P Gaffo E Zappaterra M Bortoluzzi S Davoli R. Transcriptional profiling of subcutaneous adipose tissue in Italian Large White pigs divergent for backfat thickness. Anim Genet. (2016) 47:306–23. 10.1111/age.12413 26931818

[37] Booth AD Magnuson AM Fouts J Wei Y Wang D Pagliassotti MJ. Subcutaneous adipose tissue accumulation protects systemic glucose tolerance and muscle metabolism. Adipocyte. (2018) 7:261–72. 10.1080/21623945.2018.1525252 30230416 PMC6768251

[38] Lattanzio R Piantelli M Falasca M. Role of phospholipase C in cell invasion and metastasis. Adv Biol Regul. (2013) 53:309–18. 10.1016/j.jbior.2013.07.006 23925006

[39] Sicart A Katan M Egea G Sarri E. PLCgamma1 participates in protein transport and diacylglycerol production triggered by cargo arrival at the Golgi. Traffic. (2015) 16:250–66. 10.1111/tra.12246 25491205

[40] Frigolet ME Torres N Tovar AR. The renin-angiotensin system in adipose tissue and its metabolic consequences during obesity. J Nutr Biochem. (2013) 24:2003–15. 10.1016/j.jnutbio.2013.07.002 24120291

[41] Krishnapuram R Kirk-Ballard H Dhurandhar EJ Dubuisson O Messier V Rabasa-Lhoret R. Insulin receptor-independent upregulation of cellular glucose uptake. Int J Obes. (2013) 37: 146–53. 10.1038/ijo.2012.6 PMC4841456 22310476

[42] Frank M Sodin-Semrl S Irman S Bozic B Rozman B. Beta2-glycoprotein I and annexin A5 phospholipid interactions: artificial and cell membranes. Autoimmun Rev. (2009) 9:5–10. 10.1016/j.autrev.2009.02.025 19232551

[43] Lin YC Chipot C Scheuring S. Annexin-V stabilizes membrane defects by inducing lipid phase transition. Nat Commun. (2020) 11:230. 10.1038/s41467-019-14045-w 31932647 PMC6957514

[44] Urtatiz O Van Raamsdonk CD. Gnaq and gna11 in the endothelin signaling pathway and melanoma. Front Genet. (2016) 7:59. 10.3389/fgene.2016.00059 27148356 PMC4837292

[45] Ding M Jiang S Miao J Pan L. Possible roles of gonadotropin-releasing hormone (GnRH) and melatonin in the control of gonadal development of clam Ruditapes philippinarum. Comp Biochem Physiol A Mol Integr Physiol. (2021) 262:111059. 10.1016/j.cbpa.2021.111059 34455085

[46] Fox CS Massaro JM Hoffmann U Pou KM Maurovich-Horvat P Liu CY. Abdominal visceral and subcutaneous adipose tissue compartments: association with metabolic risk factors in the Framingham Heart Study. Circulation. (2007) 116:39–48. 10.1161/CIRCULATIONAHA.106.675355 17576866

[47] Berry DC Stenesen D Zeve D Graff JM. The developmental origins of adipose tissue. Development. (2013) 140:3939–49. 10.1242/dev.080549 24046315 PMC3775412

[48] Delosière M Pires J Bernard L Cassar-Malek I Bonnet M. Milk proteome from in silico data aggregation allows the identification of putative biomarkers of negative energy balance in dairy cows. Sci Rep. (2019) 9:9711–8. 10.1038/s41598-019-46142-7 31273261 PMC6609625

[49] Casal A Garcia-Roche M Navajas EA Cassina A Carriquiry M. Hepatic mitochondrial function in Hereford steers with divergent residual feed intake phenotypes. J Anim Sci. (2018) 96:4431–43. 10.1093/jas/sky285 30032298 PMC6162574

[50] Romao JM He ML McAllister TA Guan LL. Effect of age on bovine subcutaneous fat proteome: molecular mechanisms of physiological variations during beef cattle growth. J Anim Sci. (2014) 92:3316–27. 10.2527/jas.2013-7423 24894005

[51] Wang M Li Y Gao Y Li Q Cao Y Shen Y. Vitamin E regulates bovine granulosa cell apoptosis via NRF2-mediated defence mechanism by activating PI3K/AKT and ERK1/2 signalling pathways. Reprod Domest Anim. (2021) 56:1066–84. 10.1111/rda.13950 33978262

