# Supplementary figures and images for: Identification of Key Genes Associated With Early Calf-Hood Nutrition in Subcutaneous and Visceral Adipose Tissues by Co-Expression Analysis
Source: Front Vet Sci. 2022 May 10;9:831129. doi: 10.3389/fvets.2022.831129 (PMC9127810; doi:10.3389/fvets.2022.831129)

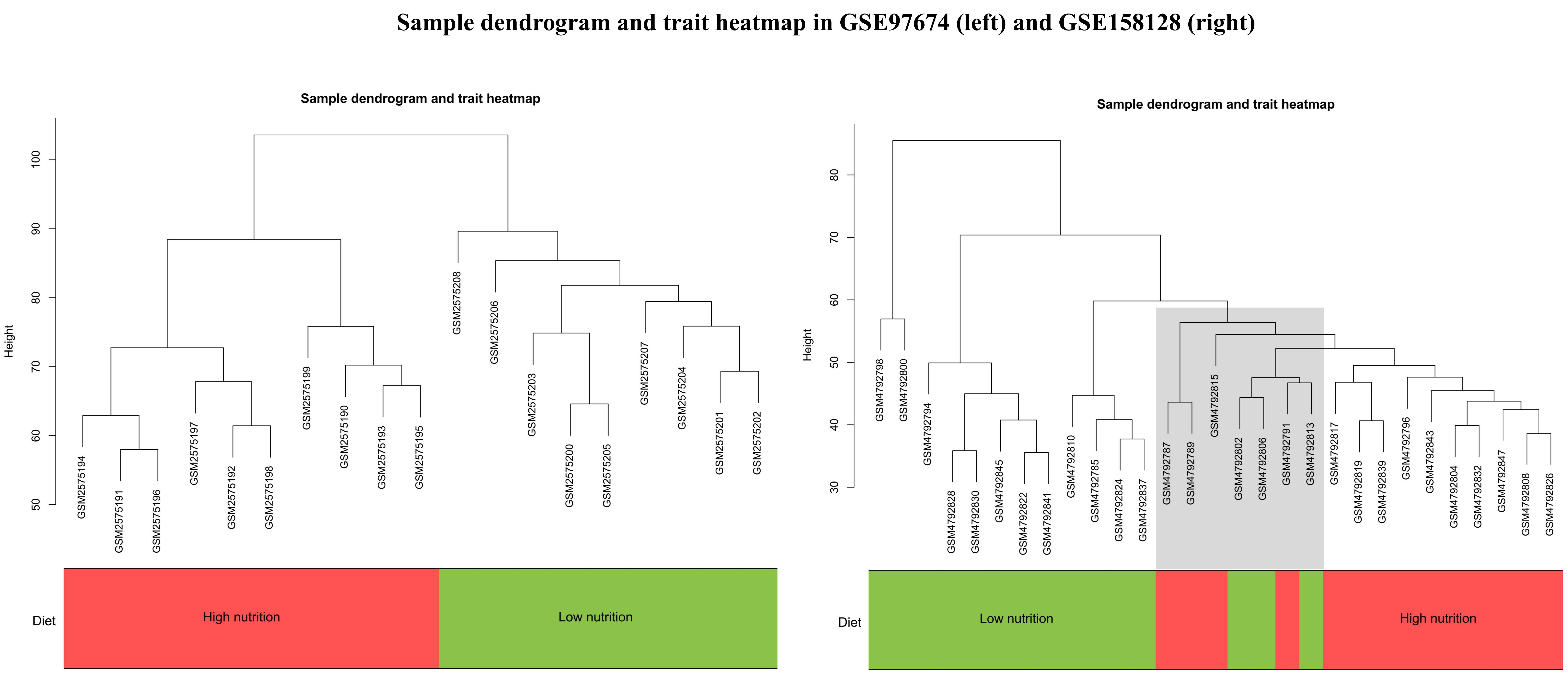

Supplement: Supplementary Figure 1 — Sample dendrogram and trait heatmap in PRJNA382633 (left) and PRJNA664093 (right). [file Image_1.JPEG]

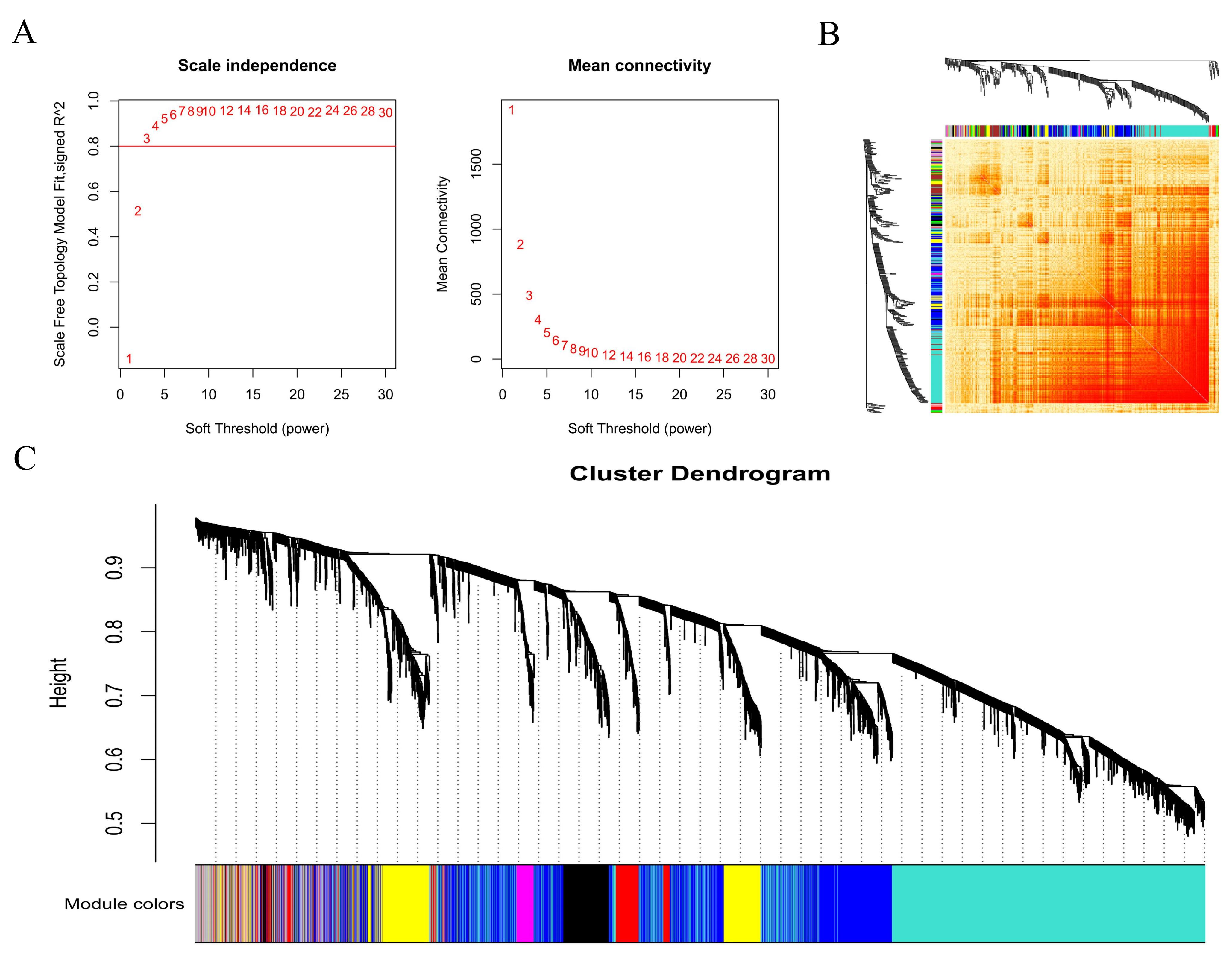

Supplement: Supplementary Figure 2 — Gene co-expression networks in subcutaneous adipose tissue (PRJNA382633). (A) Analysis of the scale-free fit index for soft-thresholding powers (left) and the mean connectivity for various soft-thresholding powers (right); (B) Network heatmap plot in the co-expression modules. (C) Gene clustering dendrogram and module identification. [file Image_2.JPEG]

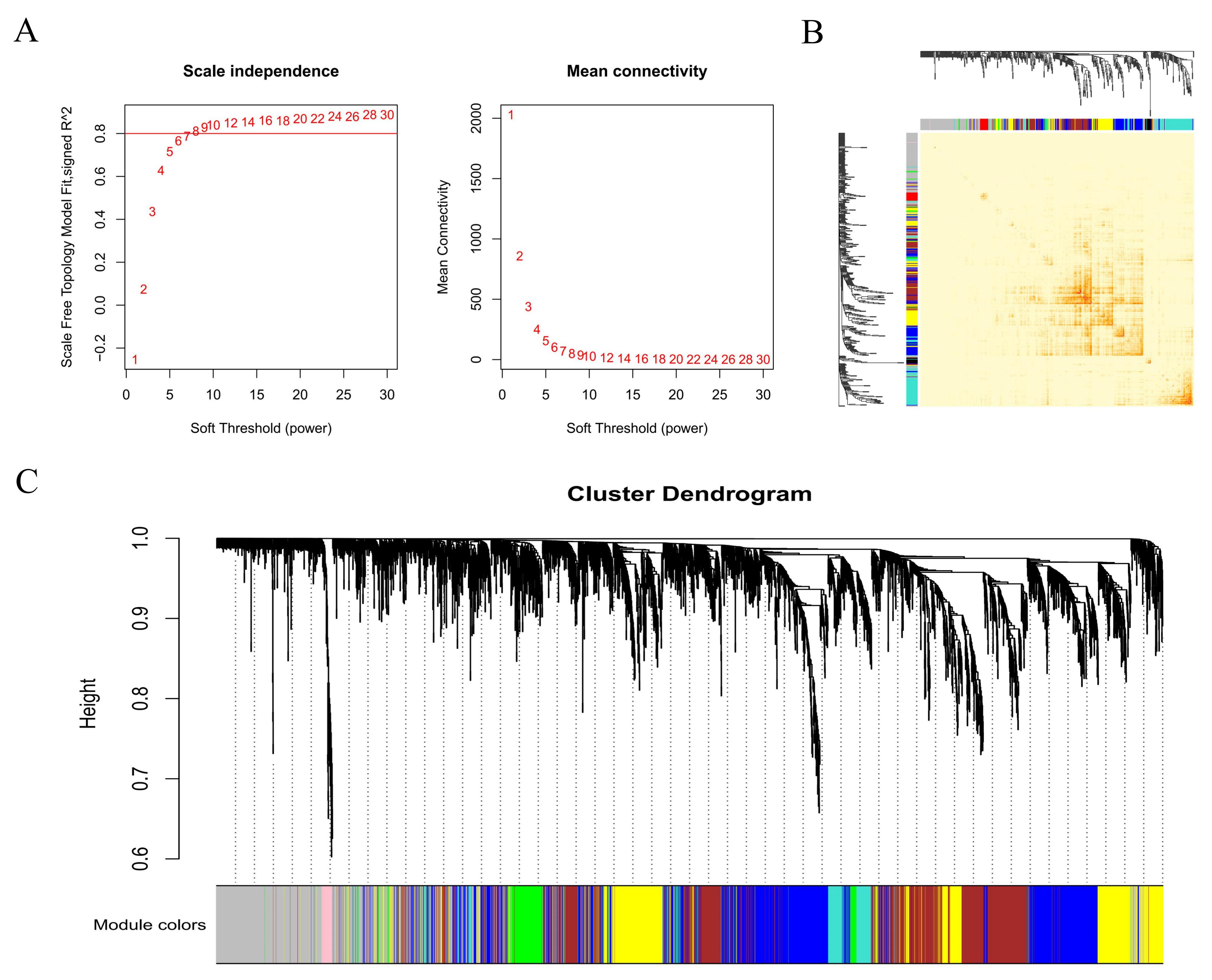

Supplement: Supplementary Figure 3 — Gene co-expression networks in visceral adipose tissue (PRJNA664093). (A) Analysis of the scale-free fit index for soft-thresholding powers (left) and the mean connectivity for various soft-thresholding powers (right); (B) Network heatmap plot in the co-expression modules. (C) Gene clustering dendrogram and module identification. [file Image_3.JPEG]

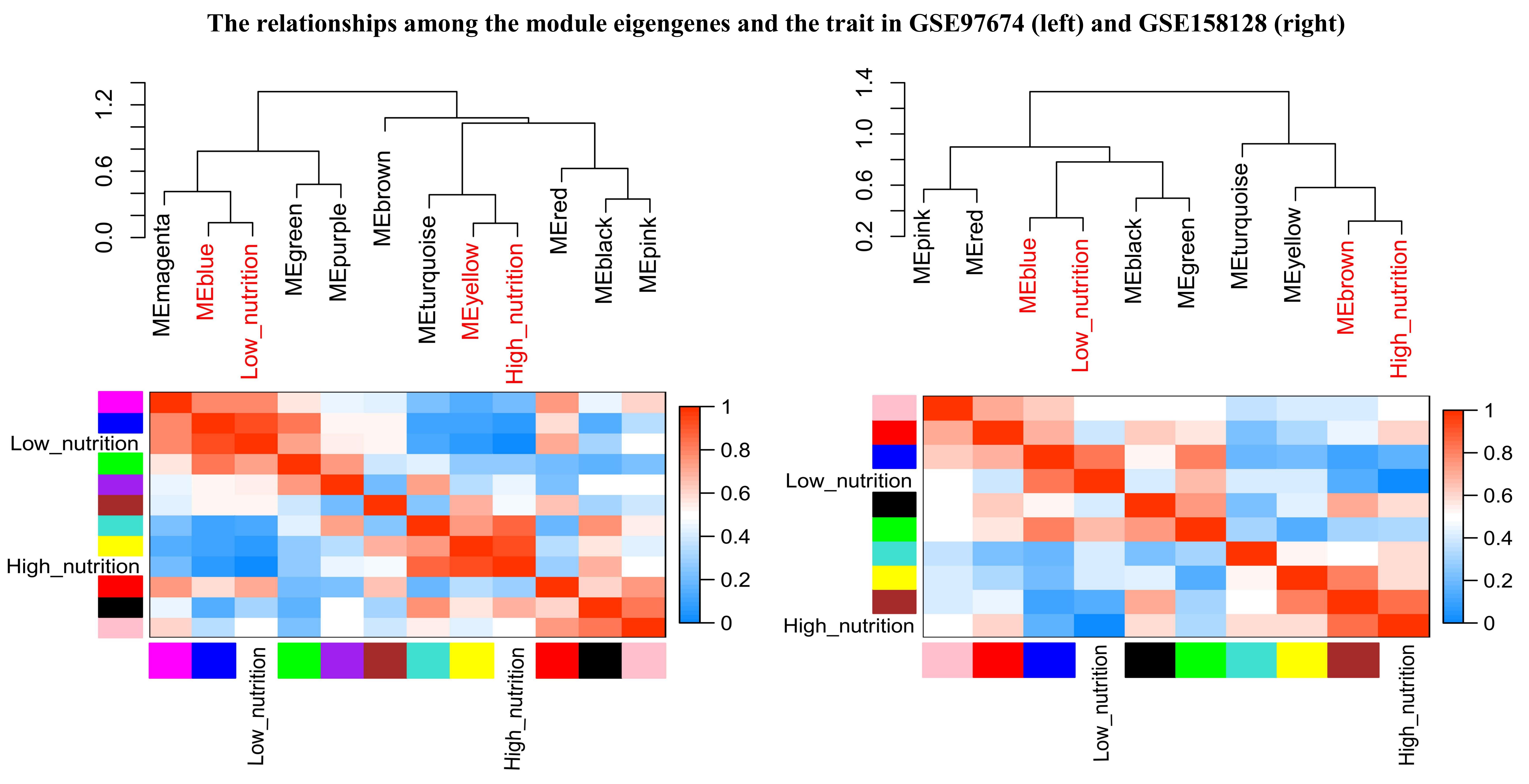

Supplement: Supplementary Figure 4 — The relationships among the module eigengenes and nutritional levels in subcutaneous adipose tissue (left) and visceral adipose tissue (right). [file Image_4.JPEG]

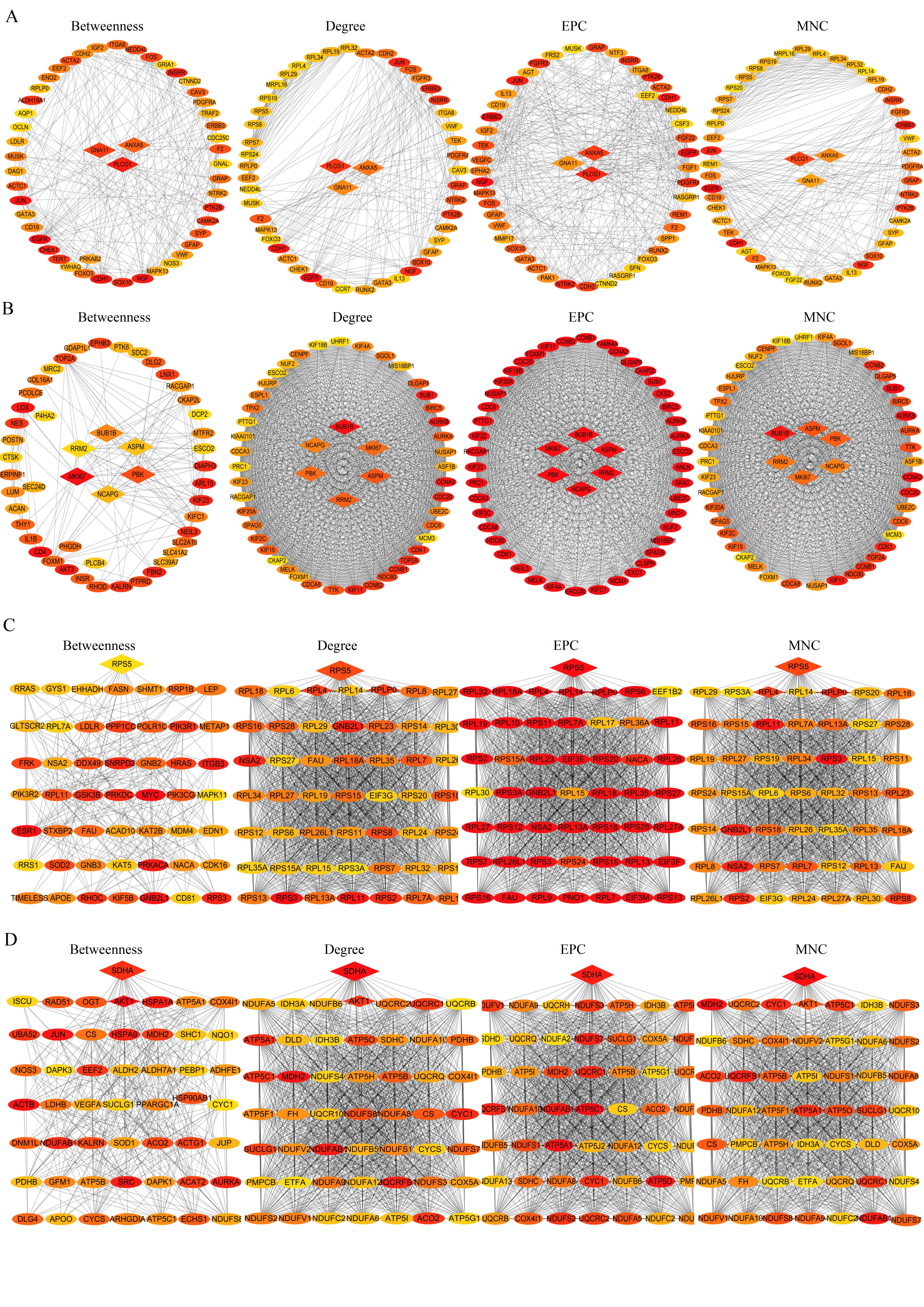

Supplement: Supplementary Figure 5 — Top 50 hub genes identified by Betweenness, Degree, EPC, and MNC in the blue (A) and yellow (B) module of dataset PRJNA382633 (subcutaneous adipose tissue), and blue (C) and brown (D) module of dataset PRJNA664093 (visceral adipose tissue). [file Image_5.JPEG]

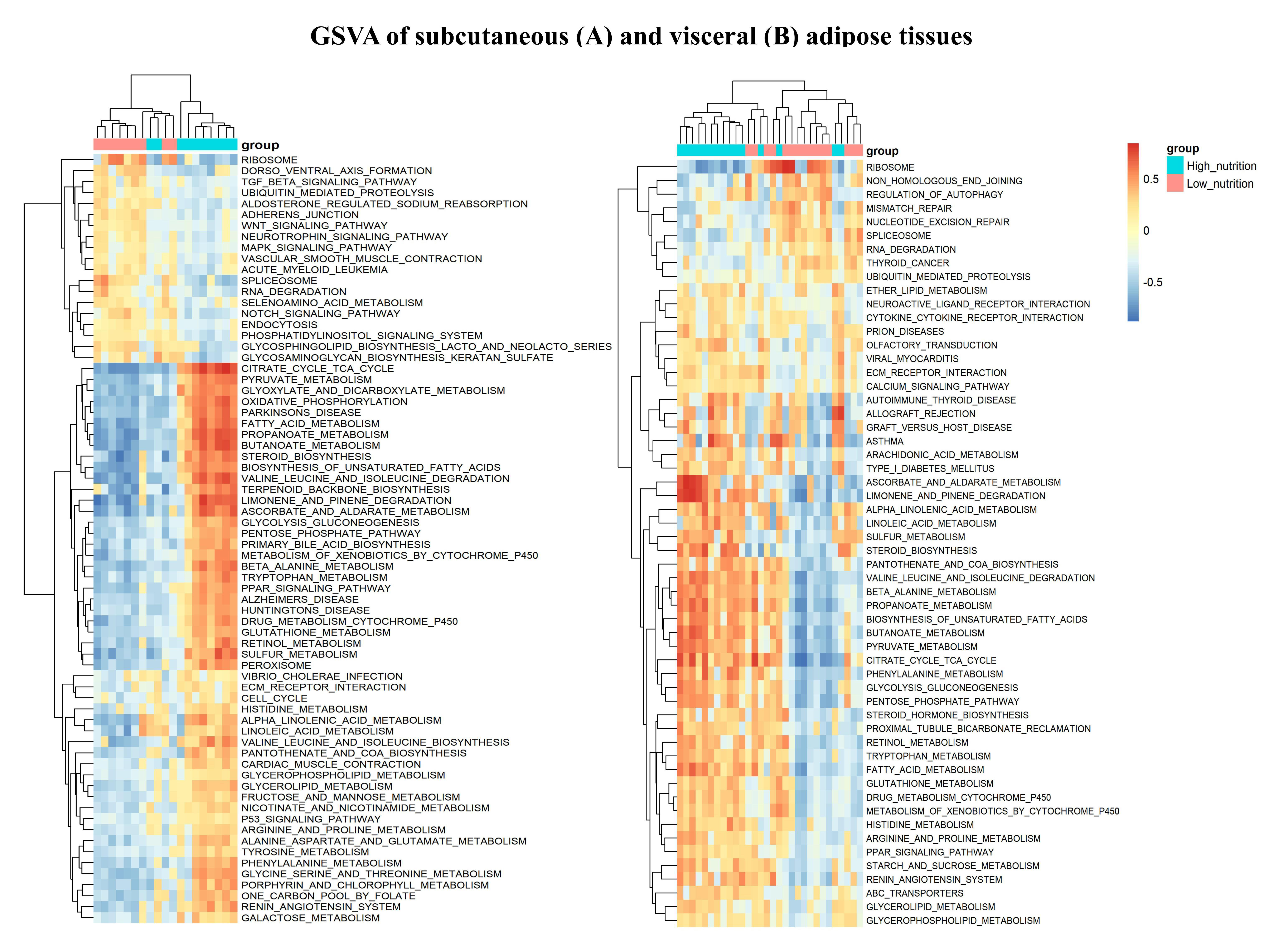

Supplement: Supplementary Figure 6 — Gene set variation analysis (GSVA) results in subcutaneous (A) and visceral (B) adipose tissues. [file Image_6.JPEG]

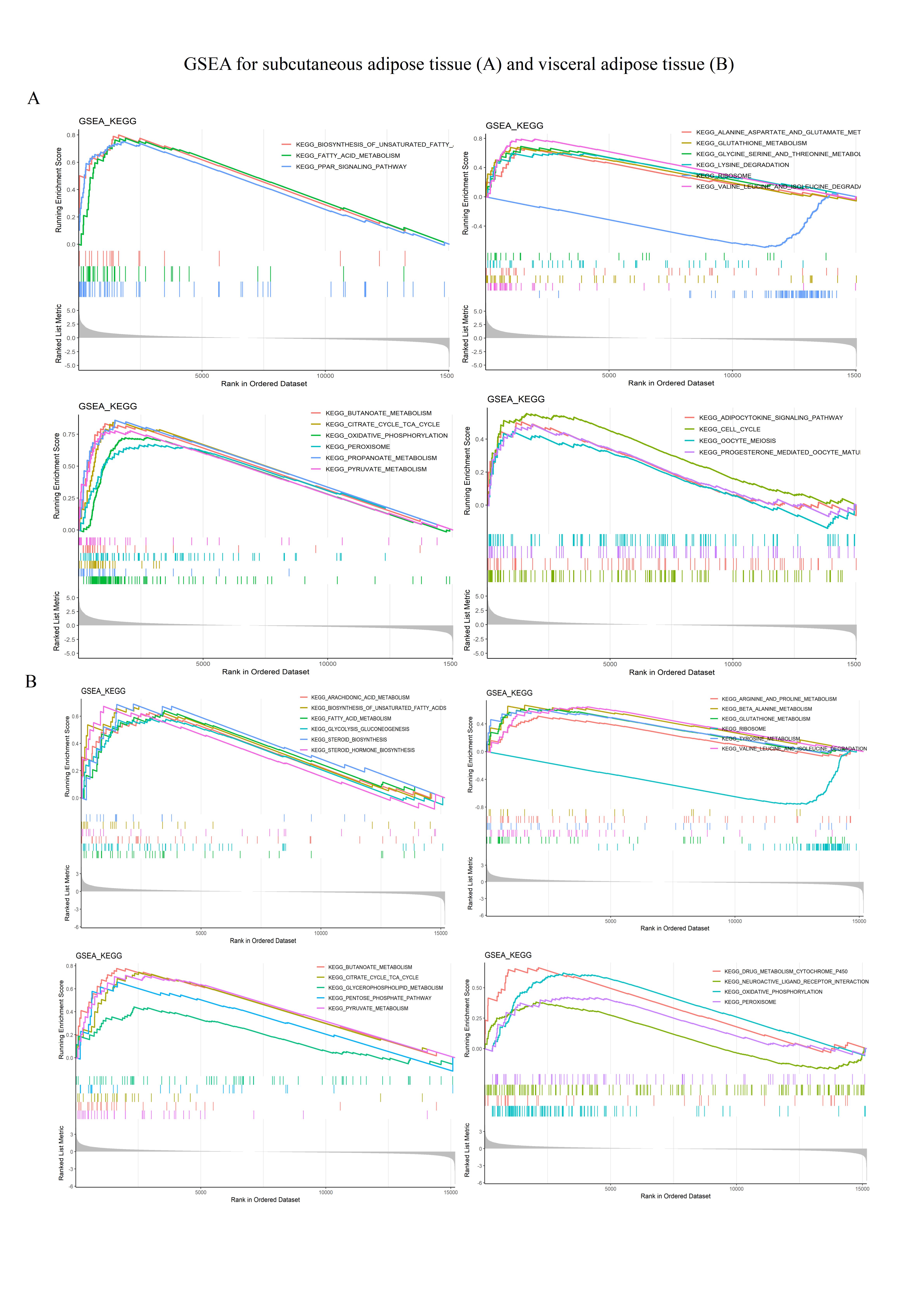

Supplement: Supplementary Figure 7 — Gene set enrichment analysis (GSEA) results in subcutaneous adipose tissue (A), and visceral adipose tissue (B). The horizontal axis represents the ranked gene list according to the log2 FC values between high and low nutrition groups. The vertical axis represents enrichment scores (upper) and the log2 FC values (lower). Each color represents a pathway marked in the upper right corner. [file Image_7.JPEG]

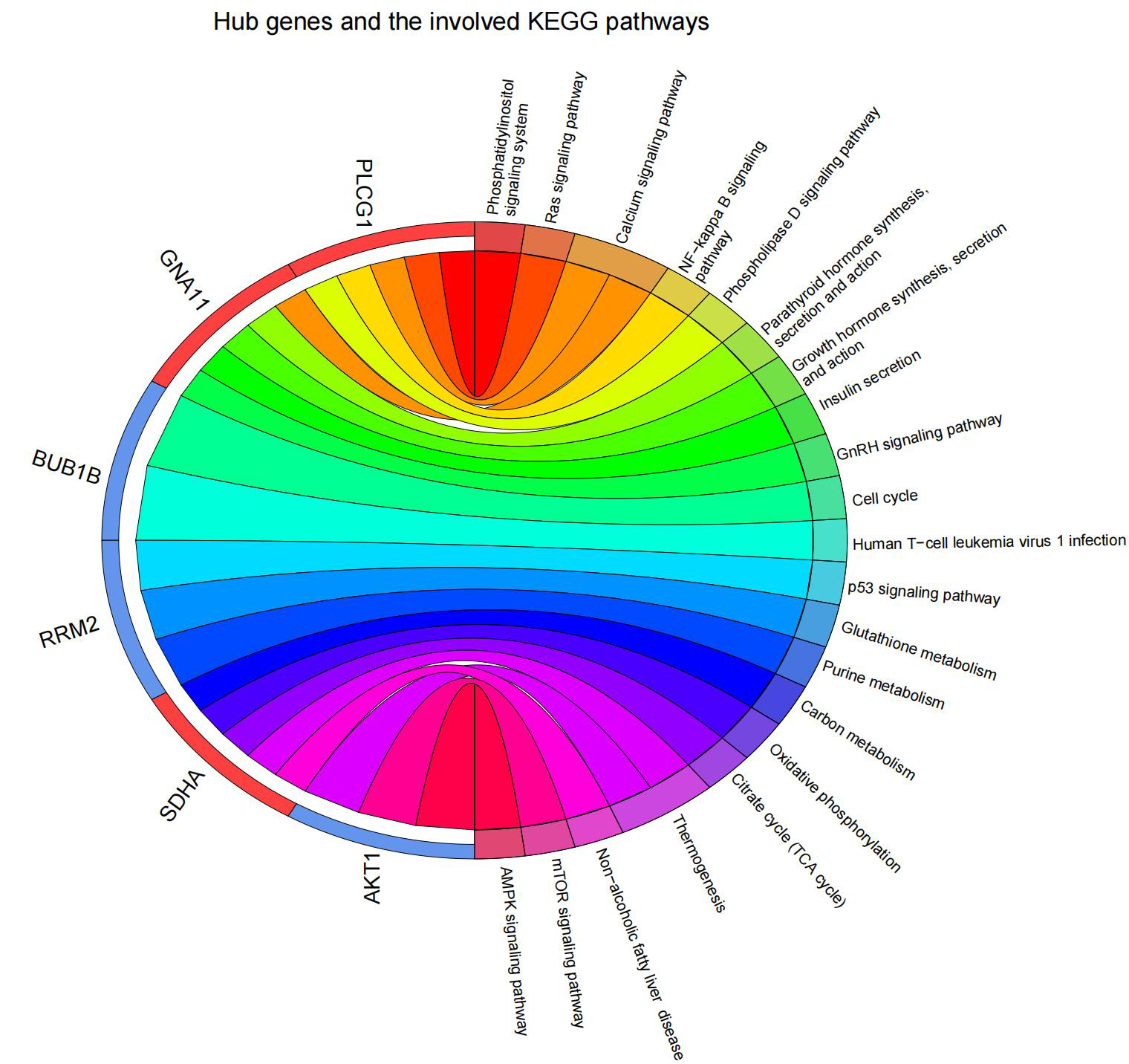

Supplement: Supplementary Figure 8 — Circos plot to indicate the relationship between hub genes and KEGG pathways. [file Image_8.JPEG]

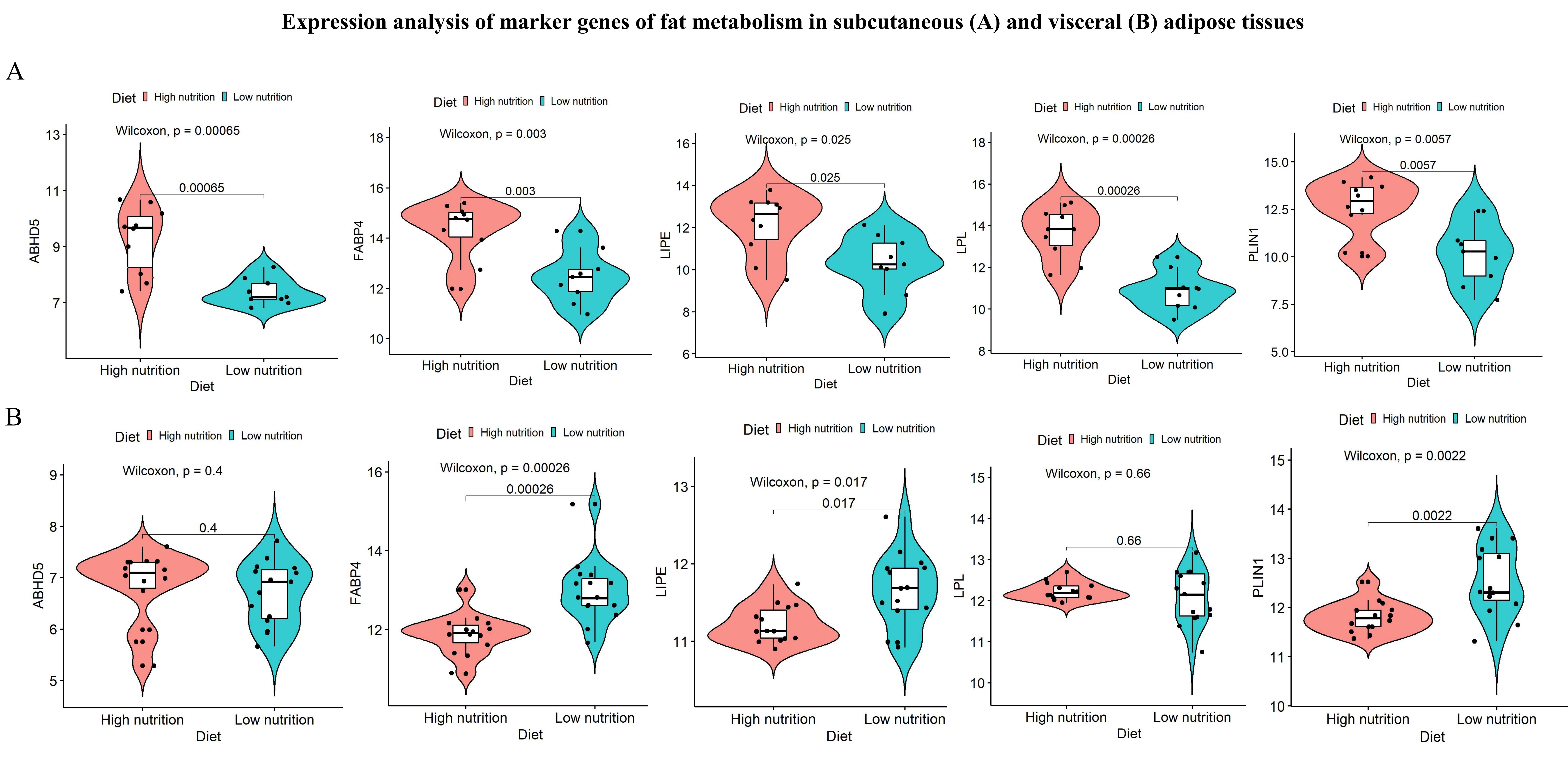

Supplement: Supplementary Figure 9 — Expression analysis of marker genes of fat metabolism in subcutaneous (A) and visceral (B) adipose tissues. [file Image_9.JPEG]
